# Supplementary material for: Fadraciclib (CYC065), a novel CDK inhibitor, targets key pro-survival and oncogenic pathways in cancer
Source: PLoS One. 2020 Jul 9;15(7):e0234103. doi: 10.1371/journal.pone.0234103 (PMC7347136; doi:10.1371/journal.pone.0234103)
Supplement: S1 Table — The cell lines utilised in this study are listed alongside various parameters of interest as indicated. Key: y = years, M = male, F = female, PB = peripheral blood, BM = bone marrow, h = hours, TNBC = triple negative breast cancer. EMT = epithelial-mesenchymal transition. Molecular genetic data listed for the AML cell lines was derived from COSMIC database (http://cancer.sanger.ac.uk/cancergenome/projects/cosmic/) or on the DSMZ website. (DOCX) [file pone.0234103.s001.docx]

**S1 Table**

**(A) AML cell line information**.

Key: y = years; M = male; F = female; PB = peripheral blood; BM = bone marrow; h = hours. Molecular genetic data listed for the individual cell lines derived from COSMIC database (http://cancer.sanger.ac.uk/cancergenome/projects/cosmic/) or on the DSMZ website

| Cell line | Type | Origin | Supplier | Doubling time (h) | Culture Media | MLL status | Molecular genetics |
| --- | --- | --- | --- | --- | --- | --- | --- |
|  |  |  |  |  |  |  |  |
| EOL-1 | AML | 33y M, PB | DSMZ | 60 | RPMI | MLL-PTD | MLLr, FIP1L1-PDGFRA fusion |
| ML-2 | AML M4 | 26y M, PB | DSMZ | 60 | RPMI | MLL-AF6 | MLLr, CDKN2A, KRAS, NOTCH1 |
| MOLM-13 | AML M5a | 20y M, PB | DSMZ | 50 | RPMI | MLL-AF9 | MLLr, FLT3 ITD |
| MV4-11 | AML M5a | 10y M, PB | DSMZ | 50 | RPMI | MLL-AF4 | MLLr, FLT3 ITD |
| NOMO-1 | AML M5a | 31y F, BM | DSMZ | 35 | RPMI | MLL-AF9 | CDKN2A, KRAS, TP53 |
| OCI-AML2 | AML M4 | 65y M, PB | DSMZ | 30-50 | a-MEM | MLL PTD | DNMT3A |
| THP-1 | AML M5 | 1y M, PB | ECAC | 35-50 | RPMI | MLL-AF9 | CDKN2A, KDM6A, NRAS, TP53 |
| HEL | AML M6 | 30y M, PB | DSMZ | 36 | RPMI | WT | CDKN2A, JAK2, TP53 |
| HL60 | AML | 35y F, PB | ATCC | 40 | RPMI | WT | CDKN2A, NRAS, TP53 |
| Kasumi-1 | AML M2 | 7y M, PB | DSMZ | 48-72 | RPMI | WT | TP53, t(8,21) translocation - RUNX1-RUNX1T1 (AML1-ETO) fusion gene |
| KG-1 | AML | 51y M, BM | DSMZ | 38 | RPMI | WT | TP53 |
| OCI-AML5 | AML M4 | 77y M, PB | DSMZ | 30-50 | a-MEM | WT | - |
| PL21 | AML | 24y M, PB | DSMZ | 50 | RPMI | WT | FLT3 ITD |

(B) Solid tumour and non-malignant cell lines used in this study

Cell lines are classified by origin. The TNBC cell lines were classified as basal or further classified into those cells that had undergone an epithelial-mesenchymal transition.

| Cell Line | Cell Line Description | Supplier | Culture Media |
| --- | --- | --- | --- |
| Colo205 | colon adenocarcinoma | ECACC | RPMI + 10% FBS + Pen/Strep |
| Cal51 | Basal-like/Post-EMT TNBC | DSMZ | DMEM + 10% FBS + Pen/Strep |
| MDA-MB-468 | Basal-like TNBC | ATCC | DMEM + 10% FBS + Pen/Strep |
| HCC1954 | HER2 positive breast cancer | ATCC | RPMI + 10% FBS + Pen/Strep |
| MCF10A | Non-malignant breast | ATCC | MEGM kit (Lonza) + supplements + 100ng/ml cholera toxin (Sigma) |
| 184A1 | Non-malignant breast | ATCC | MEGM kit (Lonza) + supplements + 0.005mg/ml Transferrin + 1ng/ml cholera toxin |
