# Supplementary material for: Fadraciclib (CYC065), a novel CDK inhibitor, targets key pro-survival and oncogenic pathways in cancer
Source: PLoS One. 2020 Jul 9;15(7):e0234103. doi: 10.1371/journal.pone.0234103 (PMC7347136; doi:10.1371/journal.pone.0234103)
Supplement: S2 Table — Antibodies are listed together with the commercial source, catalogue number, dilution used in the study and the species. (DOCX) [file pone.0234103.s002.docx]

**S2 Table**

**Antibodies used for Western Blotting**

| **Antibody** | **Source** | **Dilution** | **Secondary antibody** |
| --- | --- | --- | --- |
| Total RNA polymerase II | Covance, MMS-126R-1000 | 1:1000 | Mouse |
| RNA polymerase II pSer2 | Covance, MMS-129R-500 | 1:1000 | Mouse |
| RNA polymerase II pSer5 | Covance, MMS-134R-500 | 1:1000 | Mouse |
| Mcl-1 (Y37) | Abcam ab32087 | 1:1000 | Rabbit |
| cleaved PARP (Asp214) | Cell signaling 5625 | 1:1000 | Rabbit |
| BCL2 (clone 100) | Millipore | 1:2000 | Mouse |
| BCL-XL (BCL2L1) | Santa Cruz sc-23958 | 1:500 | Mouse |
| Bak | Calbiochem AM03-100ug | 1:40 | Mouse |
| Bax | NT-Millipore 06-499 | 1:1000 | Rabbit |
| BID | Cell Signalling 2002 | 1:1000 | Rabbit |
| BAD | Santa Cruz sc-8044 | 1:500 | Mouse |
| Hoxa9 | Abcam ab140631 | 1:1000 | Rabbit |
| Meis1 | Abcam ab19867 | 1:340 | Rabbit |
| c-MYC | Cell Signalling 9402 | 1:1000 | Rabbit |
| CDA | Abcam ab82347 | 1:100 | Rabbit |
| ENT1 | Abcam ab48607 | 1:350 | Rabbit |
| DCK | Abcam ab96599 | 1:2000 | Rabbit |
| Actin | Sigma, A1978 | 1:20000 | Mouse |
| Rabbit anti-mouse IgG HRP | Pierce, 31450 | 1:2000 | NA |
| Goat anti-rabbit IgG HRP | Sigma, A0545 | 1:10000 | NA |
