# Supplementary material for: Fadraciclib (CYC065), a novel CDK inhibitor, targets key pro-survival and oncogenic pathways in cancer
Source: PLoS One. 2020 Jul 9;15(7):e0234103. doi: 10.1371/journal.pone.0234103 (PMC7347136; doi:10.1371/journal.pone.0234103)
Supplement: S3 Table — IC50 ± SD (μM) for seliciclib, CCT068127 and fadraciclib (CYC065) for CDK2, CDK4, CDK7 and CDK9 showing values presented in Fig 1B. (DOCX) [file pone.0234103.s003.docx]

**S3 Table**

Comparison of CDK profile of seliciclib, CCT068127 and fadraciclib (CYC065)

| **Kinase** | **Seliciclib IC_50_ ± SD (µM)** | **CCT068127 IC_50_ ± SD (µM)** | **Fadraciclib (CYC065) IC_50_ ± SD (µM)** |
| --- | --- | --- | --- |
| CDK2/cyclin E | 0.42 ± 0.08 | 0.02 ± 0.002 | 0.02 ± 0.001 |
| CDK9/cyclin T1 | 2.03 ± 0.35 | 0.10 ± 0.03 | 0.10 ± 0.02 |
| CDK7/cyclin H1/MAT1 | 0.55 ± 0.12 | 0.62 ± 0.04 | 0.73 ± 0.16 |
| CDK4/cyclin D3 | 5.59 ± 0.05 | 4.20 ± 0.34 | > 10 |
