# Supplementary material for: Fadraciclib (CYC065), a novel CDK inhibitor, targets key pro-survival and oncogenic pathways in cancer
Source: PLoS One. 2020 Jul 9;15(7):e0234103. doi: 10.1371/journal.pone.0234103 (PMC7347136; doi:10.1371/journal.pone.0234103)
Supplement: S4 Table — Colo205 72 h continuous treatment IC50 ± SD (μM) for seliciclib, CCT068127, fadraciclib (CYC065) and alvocidib (flavopiridol). Values are the mean of 3 independent experiments, each run in triplicate. Values determined were used to select treatment conditions for western blotting and flow cytometry analysis shown in Fig 1. (DOCX) [file pone.0234103.s004.docx]

**S4 Table**

Colo205 IC_50_ values for seliciclib, CCT068127, fadraciclib (CYC065) and alvocidib (flavopiridol).

| **Compound** | **Colo205** **IC_50_ ± SD (µM)** |
| --- | --- |
| Seliciclib | 13.3 ± 1.3 |
| CCT068127 | 0.82 ± 0.17 |
| Fadraciclib (CYC065) | 0.31 ± 0.03 |
| Alvocidib (flavopiridol) | 0.13 ± 0.02 |
