# Supplementary figures and images for: Fadraciclib (CYC065), a novel CDK inhibitor, targets key pro-survival and oncogenic pathways in cancer
Source: PLoS One. 2020 Jul 9;15(7):e0234103. doi: 10.1371/journal.pone.0234103 (PMC7347136; doi:10.1371/journal.pone.0234103)

**S5 Table**


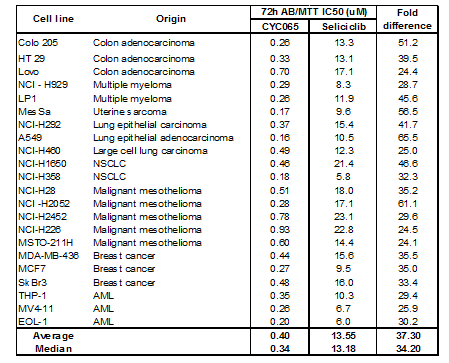

Supplement: S5 Table — The cell lines included in this study are listed along with the IC50 (μM) for seliciclib and fadraciclib (CYC065) after a continuous 72 h treatment. The fold difference in potency between seliciclib and fadraciclib (CYC065) is indicated on the right column. (DOCX) [file pone.0234103.s005.docx]

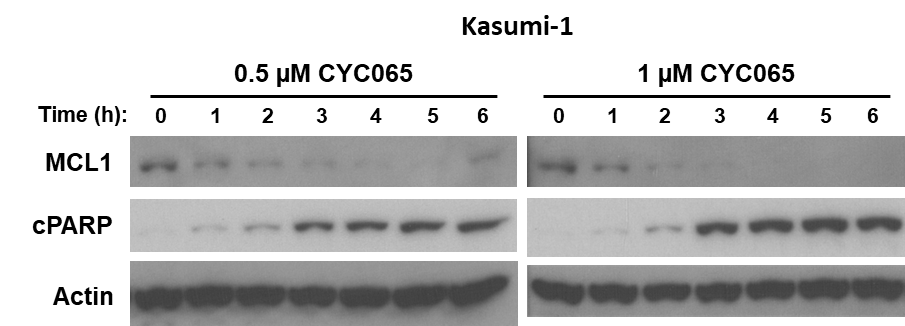
**Supplementary Figure 1A**

**Supplementary Figure 1B**


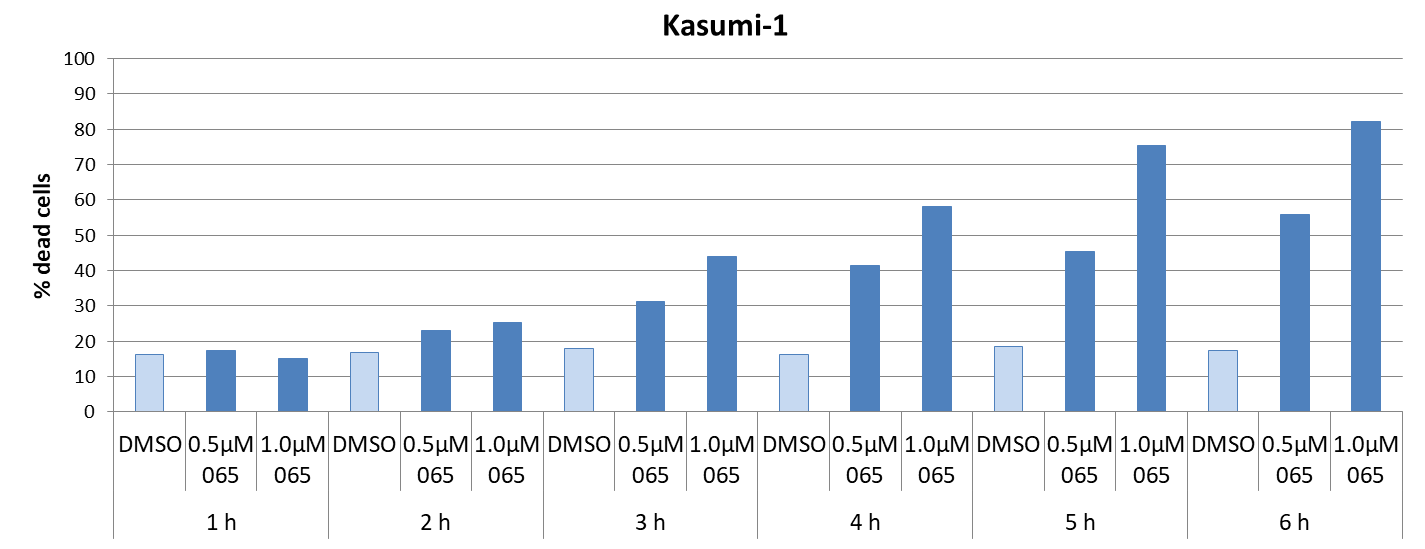

Supplement: S1 Fig — Kasumi-1 cells were treated with 0.5 or 1.0 μM fadraciclib (CYC065) for up to 6 h, with cells harvested every hour for examination of the levels of MCL1 and cleaved PARP by Western blotting (A). Kasumi-1 cells were pulse treated with 0.5 or 1.0 μM fadraciclib (CYC065) for up to 6 h with medium replaced at the indicated times, and then samples harvested at 24 h from the start of treatment to assess viability by Viacount assay (B). (DOCX) [file pone.0234103.s009.docx]

**Supplementary Figure 4**


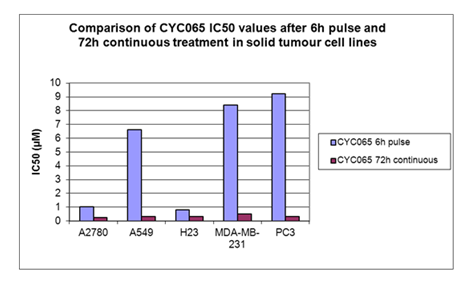

Supplement: S4 Fig — Sensitive cell lines, A2780 and H23, show similar IC50 values with short pulse or continuous treatment, whereas, the resistant cell lines A549, MDA-MB-231 and PC3, are only sensitive to prolonged treatment with fadraciclib (CYC065). (DOCX) [file pone.0234103.s012.docx]

**Supplementary Figure 5**


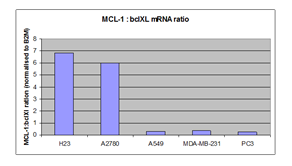

Supplement: S5 Fig — The levels of MCL1 and BCL2L1 mRNA as determined by qPCR were examined in selected sensitive and resistant solid tumour cell lines. Sensitive cell lines, H23 and A2780, had high levels of MCL1 and lower levels of BCL2L1 –confirming the results obtained by Western blotting. (DOCX) [file pone.0234103.s013.docx]

**Supplementary Figure 6**


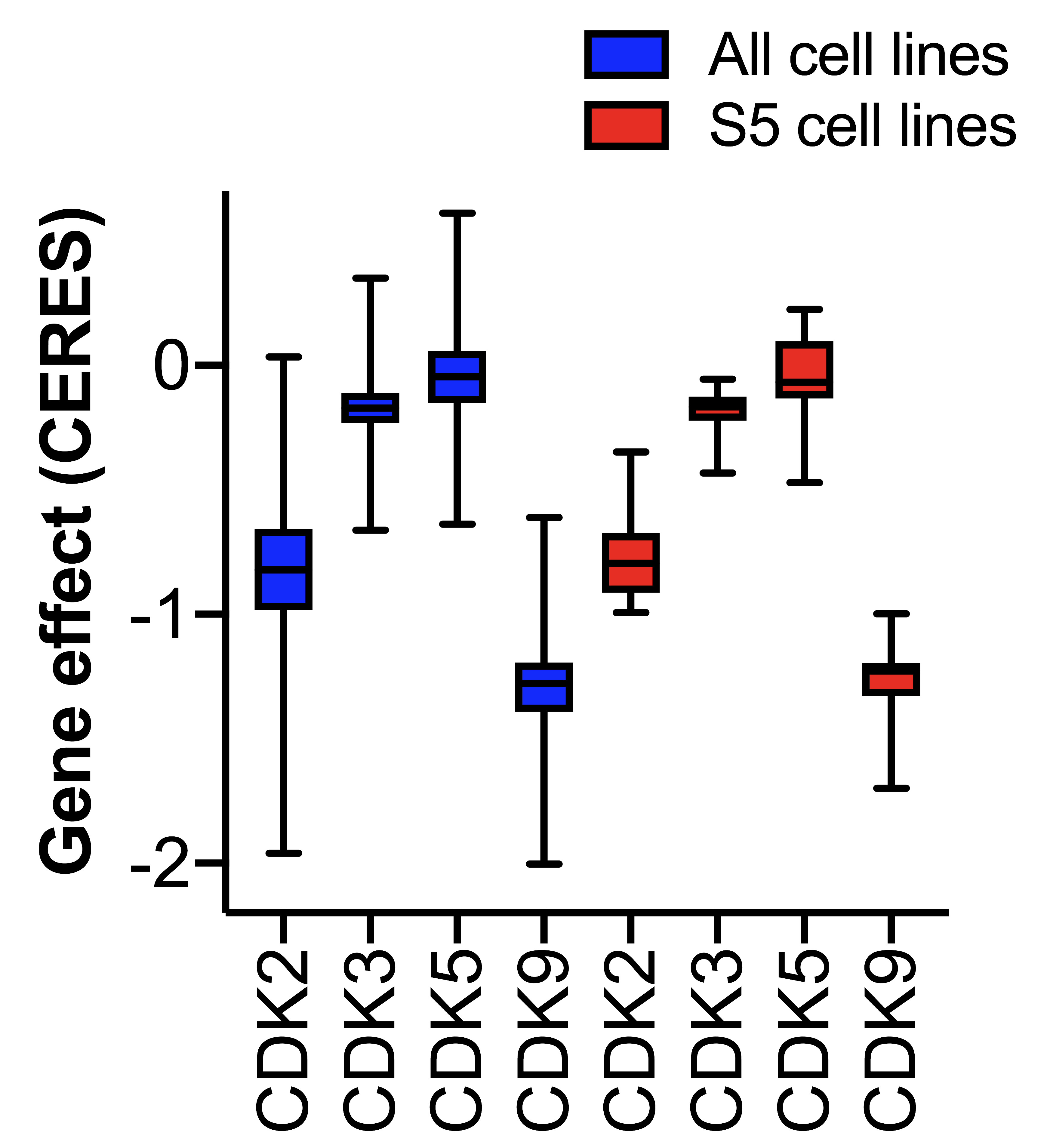

Supplement: S6 Fig — Data were obtained from genome-wide CRISPR-Cas9 screens with the Avana sgRNA library in cancer cell lines and deposited as part of the Cancer Dependency Map project (https://depmap.org/portal/; Computational correction of copy number effect improves specificity of CRISPR–Cas9 essentiality screens in cancer cells [72]. A lower CERES score indicates a higher likelihood that the gene of interest is essential in a given cell line. The blue box-whisker plots correspond to data for all of the >700 cancer cells for which data are available in DepMap; the red box-whisker plots correspond to data from cancer cell lines described in S5 Table. The box shows the median value and the interquartile range between the first and third quartiles. The bars show the minimum and maximum range of the population. (DOCX) [file pone.0234103.s014.docx]
