# Supplementary material for: Fadraciclib (CYC065), a novel CDK inhibitor, targets key pro-survival and oncogenic pathways in cancer
Source: PLoS One. 2020 Jul 9;15(7):e0234103. doi: 10.1371/journal.pone.0234103 (PMC7347136; doi:10.1371/journal.pone.0234103)
Supplement: S6 Table — Fadraciclib (CYC065) (1μM) was evaluated in a 256-kinase panel at approximately Km[ATP] and showed excellent selectivity. The percent inhibition of each kinase by fadraciclib (CYC065) is indicated in the table. Nine kinases were inhibited by >50% and the IC50 values were established against these CDK and CDK-like kinases in a separate assay. (DOCX) [file pone.0234103.s006.docx]

**S6 Table**

**Carna Bioscience kinase profiler screening**. 1 µM fadraciclib (CYC065) screened in a 256-kinase panel at around Km[ATP]

| Kinase | % Inhibition ± SD | Kinase | % Inhibition ± SD | Kinase | % Inhibition ± SD |
| --- | --- | --- | --- | --- | --- |
| ABL | -12.7 ± 0.3 | FES | 14.9 ± 1.1 | SRM | 3.5 ± 0.1 |
| ABL[T315I] | -13.0 ± 0.1 | FGFR1 | 7.8 ± 0.0 | YES | -7 ± 0.1 |
| ARG | 1.8 ± 0.1 | FGFR2 | -17.6 ± 2.2 | SYK | 2.2 ± 0.1 |
| ACK | -0.7 ± 0.0 | FGFR3 | -5.6 ± 0.2 | ZAP70 | -11.6 ± 0.3 |
| TNK1 | -0.2 ± 0.2 | FGFR4 | -14.8 ± 0.0 | BMX | 6.6 ± 0.3 |
| ALK | -9.0 ± 0.3 | IGF1R | -17.1 ± 0.2 | BTK | 5.3 ± 0.4 |
| LTK | 6.6 ± 0.1 | INSR | -12.2 ± 0.8 | ITK | 2.3 ± 0.1 |
| AXL | -11.1 ± 0.1 | IRR | -5.5 ± 0.0 | TEC | -14.9 ± 0.0 |
| MER | -9.1 ± 0.1 | JAK1 | -3.1 ± 0.0 | TXK | 3.1 ± 0.1 |
| TYRO3 | -14.8 ± 0.2 | JAK2 | 6.1 ± 0.2 | TIE2 | 2.9 ± 0.2 |
| CSK | -18.7 ± 0.4 | JAK3 | -9.5 ± 0.0 | TRKA | -10.3 ± 0.3 |
| CTK | 3.4 ± 0.2 | TYK2 | -0.1 ± 0.0 | TRKB | -11.4 ± 0.4 |
| DDR2 | -9.9 ± 0.1 | MET | -8.3 ± 0.1 | TRKC | 7.2 ± 0.3 |
| EGFR | -10.8 ± 0.4 | RON | 1.6 ± 0.1 | FLT1 | 1.9 ± 0.0 |
| EGFR[T790M] | -9.6 ± 0.1 | MUSK | 11.4 ± 0.3 | FLT4 | 6.3 ± 0.2 |
| HER2 | -17.6 ± 1.0 | FLT3 | -8.7 ± 0.1 | KDR | -12.8 ± 0.1 |
| HER4 | -13.0 ± 0.3 | FMS | -6.3 ± 0.1 | AKT1 | -0.6 ± 0.0 |
| EphA1 | 5.1 ± 0.0 | KIT | -2.4 ± 0.1 | AKT2 | 1.5 ± 0.1 |
| EphA2 | 3.8 ± 0.0 | PDGFRa | 5.8 ± 0.2 | AKT3 | 2.4 ± 0.3 |
| EphA3 | 1.6 ± 0.1 | PDGFRb | 3.2 ± 0.1 | CRIK | -1.2 ± 0.0 |
| EphA4 | 1.0 ± 0.0 | RET | 3.4 ± 0.1 | ROCK1 | -1.3 ± 0.0 |
| EphA5 | 4.9 ± 0.2 | ROS | -2 ± 0.1 | ROCK2 | 4.2 ± 0.0 |
| EphA6 | 5.7 ± 0.3 | BLK | -3.6 ± 0.0 | MRCKa | -14.8 ± 0.1 |
| EphA7 | 6.0 ± 0.3 | BRK | 2.4 ± 0.1 | PKACa | -0.8 ± 0.0 |
| EphA8 | 4.2 ± 0.2 | FGR | 3.3 ± 0.1 | PRKX | -4.2 ± 0.0 |
| EphB1 | 3.9 ± 0.1 | FRK | 3.6 ± 0.0 | PGK | -21.7 ± 0.2 |
| EphB2 | 5.0 ± 0.2 | FYN | 7.4 ± 0.3 | CGK2 | -12.6 ± 0.5 |
| EphB3 | 0.0 ± 0.0 | HCK | -4.9 ± 0.1 | PDK1 | -5.4 ± 0.1 |
| EphB4 | 3.6 ± 0.1 | LCK | -7.3 ± 0.1 | PKCa | 17.8 ± 0.5 |
| FAK | 23.4 ± 2.9 | LYNa | 2.2 ± 0.1 | PKCb1 | -1.9 ± 0.0 |
| PYK2 | 11.7 ± 0.2 | LYNb | -8.7 ± 0.1 | PKCg | 17.2 ± 0.0 |
| FER | -0.3 ± 0.0 | SRC | 2.7 ± 0.0 | PKCd | 28.7 ± 2.7 |
| PKCe | 7.6 ± 0.1 | MAPKAPK3 | -6.1 ± 0.3 | IKKb | 0.2 ± 0.0 |
| PKCq | 0.7 ± 0.0 | MAPKAPK5 | -14.5 ± 0.2 | IKKe | 2.5 ± 0.1 |
| PKCz | -0.2 ± 0.0 | skMLCK | -9.8 ± 1.1 | TBK1 | -4.8 ± 0.1 |
| PKCh | -6.1 ± 0.1 | MNK1 | -13.1 ± 1.6 | NEK1 | -18.3 ± 0.1 |
| PKCi | -13.6 ± 0.5 | MNK2 | -4.3 ± 0.1 | NEK2 | -18.1 ± 0.6 |
| PKN1 | -17.7 ± 0.1 | PIM1 | 7.4 ± 0.1 | NEK6 | -16.6 ± 0.5 |
| MSK1 | 5.5 ± 0.4 | PIM2 | 2.9 ± 0.2 | NEK7 | 0.9 ± 0.0 |
| Kinase | % Inhibition ± SD | Kinase | % Inhibition ± SD | Kinase | % Inhibition ± SD |
| MSK2 | -3.7 ± 0.0 | PHKG1 | -15.0 ± 0.4 | PBK | -0.9 ± 0.0 |
| p70S6K | 5.6 ± 0.2 | PHKG2 | -10.0 ± 0.1 | PLK1 | 5.7 ± 0.3 |
| RSK1 | 5.1 ± 0.0 | PKD1 | 12.4 ± 0.5 | PLK2 | 1 ± 0.0 |
| RSK2 | 5.6 ± 0.1 | PKD2 | -13.4 ± 0.6 | PLK3 | 5.7 ± 0.3 |
| RSK3 | 7.7 ± 0.0 | PKD3 | -1.4 ± 0.0 | TTK | 25.3 ± 4.5 |
| SGK | -0.8 ± 0.0 | CHK2 | -7.6 ± 0.1 | WNK1 | -3.3 ± 0.0 |
| SGK2 | 0.9 ± 0.0 | TSSK1 | 16.0 ± 0.5 | IRAK1 | 1.6 ± 0.1 |
| SGK3 | 6.2 ± 0.1 | TSSK2 | 0.7 ± 0.0 | IRAK4 | -10.1 ± 0.1 |
| CaMK4 | -4.8 ± 0.1 | CDC2 | 57.9 ± 1.3 | BMPR1A | 2.2 ± 0.0 |
| CaMK1a | -14.8 ± 0.2 | CDK2 | 97.3 ± 0.0 | CK1a | 8.2 ± 0.3 |
| CaMK2a | -3.6 ± 0.1 | CDK3 | 95.9 ± 0.0 | CK1d | 13.8 ± 0.4 |
| AMPKa1 | -3.8 ± 0.0 | CDK4 | 75.7 ± 5.0 | CK1e | 19.8 ± 0.5 |
| BRSK1 | -11.5 ± 0.1 | CDC7 | -5.7 ± 0.0 | PAK2 | -2.9 ± 0.0 |
| BRSK2 | 1.1 ± 0.0 | CDK5 | 98.1 ± 4.0 | PAK6 | -5.4 ± 0.0 |
| CHK1 | 1.3 ± 0.0 | DYRK2 | -1.8 ± 0.1 | MINK | -15.8 ± 0.3 |
| MARK1 | -9.7 ± 0.1 | CLK1 | 70.2 ± 6.8 | MST4 | -2.1 ± 0.0 |
| MARK2 | -1.9 ± 0.1 | HIPK2 | 2.5 ± 0.1 | Erk1 | -6.5 ± 0.0 |
| MARK3 | -0.9 ± 0.1 | GSK3a | 19.3 ± 1.3 | Erk2 | 19.6 ± 0.8 |
| MELK | 5 ± 0.1 | GSK3b | 23.8 ± 1.0 | Erk5 | 3.7 ± 0.1 |
| MGC42105 | -7.3 ± 0.1 | SRPK1 | 5.9 ± 0.2 | JNK1 | -13.5 ± 1.3 |
| NuaK1 | -2.5 ± 0.0 | SRPK2 | -3.8 ± 0.1 | JNK2 | -19.3 ± 2.2 |
| PASK | -0.4 ± 0.0 | AurA | -16.2 ± 0.4 | JNK3 | 10.6 ± 0.6 |
| DAPK1 | -8.8 ± 0.1 | AurB | 4.2 ± 0.2 | p38a | -13.3 ± 0.3 |
| DCAMKL2 | -12.4 ± 0.1 | AurC | 3.0 ± 0.2 | p38b | -19 ± 0.2 |
| MAPKAPK2 | -8.6 ± 0.1 | IKKa | 0.0 ± 0.0 | p38g | -0.6 ± 0.0 |
| p38d | -16.0 ± 0.6 | BRAF[V600E] | -1.9 ± 0.1 | EEF2K | -10.3 ± 0.2 |
| MAP2K1 | -6.8 ± 0.1 | MAP4K2 | 2.0 ± 0.1 | EGFR[L858R] | 1.7 ± 0.0 |
| MAP2K2 | 4.1 ± 0.0 | HGK | -23.8 ± 2.7 | FGFR3[K650E] | 5.6 ± 0.0 |
| MAP2K3 | -12.3 ± 0.3 | PKCb2 | -14.3 ± 0.1 | FGFR3[K650M] | 3.2 ± 0.1 |
| MAP2K4 | -2.7 ± 0.1 | WEE1 | 35.1 ± 0.1 | HIPK1 | 6.8 ± 0.3 |
| MAP2K5 | 11.9 ± 1.6 | LIMK1 | 12.3 ± 0.4 | HIPK3 | -1.1 ± 0.0 |
| MAP2K6 | 0.7 ± 0.0 | PLK4 | -38.5 ± 1.0 | KIT[T670I] | -9.2 ± 0.2 |
| MAP2K7 | -10.6 ± 0.6 | CK2a1 | -22.8 ± 0.7 | KIT[V560G] | 2.5 ± 0.1 |
| MAP3K1 | -6.6 ± 0.1 | PEK | 0.3 ± 0.0 | LOK | -23.8 ± 0.3 |
| MAP3K2 | -14.7 ± 0.8 | PKR | 1.1 ±0.0 | MARK4 | -5.1 ± 0.4 |
| MAP3K3 | -2.6 ± 0.1 | AMPKa2 | -12.8 ± 0.7 | MET[Y1235D] | 2.4 ± 0.1 |
| MAP3K4 | 7.6 ± 0.1 | CaMK1d | -14.5 ± 0.1 | MST1 | 4.0 ± 0.1 |
| MAP3K5 | 5.2 ± 0.2 | CaMK2g | 12.5 ± 0.4 | MST2 | -1.5 ± 0.0 |
| TAK1 | 0.3 ± 0.0 | CDK6 | 27.0 ± 0.9 | MST3 | -16.4 ± 0.7 |
| COT | -15.6 ± 0.4 | CDK7 | 86.7 ± 10.9 | NDR1 | 2.0 ± 0.1 |
| MLK1 | -11.2 ± 0.1 | CDK9 | 96.6 ± 3.2 | NEK9 | -23.1 ± 0.2 |
| MLK2 | 0.6 ± 0.0 | CLK2 | 87.8 ± 2.0 | p70S6Kb | 1.9 ± 0.0 |
| Kinase | % Inhibition ± SD | Kinase | % Inhibition ± SD | Kinase | % Inhibition ± SD |
| MLK3 | -4.9 ± 0.4 | DDR1 | -9.3 ± 0.1 | PAK1 | -5.8 ± 0.2 |
| DLK | 0.8 ± 0.0 | DYRK1A | 5.5 ± 0.7 | PDGFRa[T674I] | -11.4 ± 0.2 |
| RAF1 | -1.1 ± 0.1 | DYRK1B | 15.5 ± 1.3 | RSK4 | 6.0 ± 0.2 |
| BRAF | -8.4 ± 1.0 | DYRK3 | 0.9 ± 0.0 | SLK | 2.5 ± 0.1 |
