# Supplementary material for: Fadraciclib (CYC065), a novel CDK inhibitor, targets key pro-survival and oncogenic pathways in cancer
Source: PLoS One. 2020 Jul 9;15(7):e0234103. doi: 10.1371/journal.pone.0234103 (PMC7347136; doi:10.1371/journal.pone.0234103)
Supplement: S7 Table — AML cell lines were incubated with fadraciclib (CYC065) for the indicated duration and IC50 values were determined, and compared. Nine out of thirteen cell lines were highly sensitive to fadraciclib (CYC065) and displayed 6 h pulse IC50 values similar to their 72 h continuous IC50 values. Values are the mean of 3 independent experiments. (DOCX) [file pone.0234103.s007.docx]

**S7 Table**

| AML cell line | Classification | Fadraciclib 6 h pulse IC_50_ ± SD (µM) | Fadraciclib 72 h continuous IC_50_ ± SD (µM) |
| --- | --- | --- | --- |
| EOL-1 | MLL-PTD | 0.35 ± 0.05 | 0.37 ± 0.05 |
| ML_2 | MLL-AF6 | 0.33 ± 0.12 | 0.19 ± 0.05 |
| MOLM-13 | MLL-AF9; flt3-ITD | 0.25 ± 0.05 | 0.24 ± 0.05 |
| MV4-11 | MLL-AF4; flt3-ITD | 0.51 ± 0.04 | 0.26 ± 0.08 |
| Nomo-1 | MLL-AF9 | 0.66 ± 0.11 | 0.29 ± 0.05 |
| OCI-AML2 | MLL-PTD; DNMT3A | 0.55 ± 0.07 | 0.22 ± 0.03 |
| THP-1 | MLL-AF9 | 1.08 ± 0.23 | 0.57 ± 0.18 |
| HEL |  | 5.11 ± 0.88 | 0.53 ± 0.20 |
| HL60 |  | 0.79 ± 0.14 | 0.44 ± 0.07 |
| Kasumi-1 | AML-ETO | 0.32 ± 0.05 | 0.23 ± 0.04 |
| KG-1 |  | 0.47 ± 0.12 | 0.35 ± 0.03 |
| OCI-AML5 |  | 1.45 ± 0.05 | 0.31 ± 0.13 |
| PL21 | Flt3-ITD | 2.54 ± 2.71 | 0.34 ± 0.09 |
