# Supplementary material for: Fadraciclib (CYC065), a novel CDK inhibitor, targets key pro-survival and oncogenic pathways in cancer
Source: PLoS One. 2020 Jul 9;15(7):e0234103. doi: 10.1371/journal.pone.0234103 (PMC7347136; doi:10.1371/journal.pone.0234103)
Supplement: S8 Table — Concomitant treatment with fadraciclib and BCL2 inhibitor venetoclax (ABT199) or BCL2/BCL2L1 inhibitors ABT263 and ABT737 was performed and analysed as described in Materials and Methods. Average combination index (CI) and SD values are listed. (DOCX) [file pone.0234103.s008.docx]

**S8 Table**

| **Combination** | **CYC065+ABT199** | | **CYC065+ABT263** | | **CYC065+ABT737** | |
| --- | --- | --- | --- | --- | --- | --- |
| **Fraction affected** | **Avg. CI** | **SD** | **Avg. CI** | **SD** | **Avg. CI** | **SD** |
| **0.5** | **0.73** | 0.39 | **0.82** | 0.18 | **0.38** | 0.04 |
| **0.75** | **0.34** | 0.16 | **0.25** | 0.08 | **0.10** | 0.04 |
| **0.9** | **0.24** | 0.09 | **0.12** | 0.01 | **0.06** | 0.01 |
