# Supplementary material for: Fadraciclib (CYC065), a novel CDK inhibitor, targets key pro-survival and oncogenic pathways in cancer
Source: PLoS One. 2020 Jul 9;15(7):e0234103. doi: 10.1371/journal.pone.0234103 (PMC7347136; doi:10.1371/journal.pone.0234103)

**Supplementary Figure 2**

**MLL status correlates with sensitivity to fadraciclib (CYC065) in ALL cell lines**


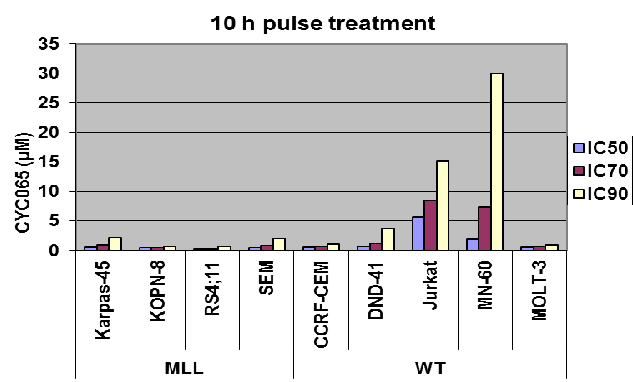

Supplement: S2 Fig — An ALL cell line panel was treated with fadraciclib (CYC065) for 10 h then incubated in compound-free medium until viability was assessed at 72 h. All cell lines with MLLr/MLL-PTD appeared to be more sensitive to fadraciclib (CYC065) than non-MLLr/MLL-PTD cell lines. (DOCX) [file pone.0234103.s010.docx]
