# Supplementary material for: Correction: Fadraciclib (CYC065), a novel CDK inhibitor, targets key pro-survival and oncogenic pathways in cancer
Source: PLoS One. 2021 May 6;16(5):e0251671. doi: 10.1371/journal.pone.0251671 (PMC8101907; doi:10.1371/journal.pone.0251671)
Supplement: S1 File — (PDF) [file pone.0251671.s001.pdf]

RESEARCH ARTICLE

Fadraciclib (CYC065), a novel CDK inhibitor, targets key pro-survival and oncogenic pathways in cancer

Sheelagh Frame[1], Chiara Saladino[1], Craig MacKay[1], Butrus Atrash[2], Peter Sheldrake[2], Edward McDonald[2], Paul A. Clarke[2], Paul Workman[2], David Blake[1], Daniella Zheleva[1]*

1 Cyclacel Limited, Dundee, United Kingdom, **2** Cancer Research UK Cancer Therapeutics Unit, Division of Cancer Therapeutics, The Institute of Cancer Research, London, United Kingdom

* daniellazheleva@yahoo.com

Abstract

Cyclin-dependent kinases (CDKs) contribute to the cancer hallmarks of uncontrolled proliferation and increased survival. As a result, over the last two decades substantial efforts have been directed towards identification and development of pharmaceutical CDK inhibitors. Insights into the biological consequences of CDK inhibition in specific tumor types have led to the successful development of CDK4/6 inhibitors as treatments for certain types of breast cancer. More recently, a new generation of pharmaceutical inhibitors of CDK enzymes that regulate the transcription of key oncogenic and pro-survival proteins, including CDK9, have entered clinical development. Here, we provide the first disclosure of the chemical structure of fadraciclib (CYC065), a CDK inhibitor and clinical candidate designed by further optimization from the aminopurine scaffold of seliciclib. We describe its synthesis and mechanistic characterization. Fadraciclib exhibits improved potency and selectivity for CDK2 and CDK9 compared to seliciclib, and also displays high selectivity across the kinome. We show that the mechanism of action of fadraciclib is consistent with potent inhibition of CDK9-mediated transcription, decreasing levels of RNA polymerase II C-terminal domain serine 2 phosphorylation, the pro-survival protein Myeloid Cell Leukemia 1 (MCL1) and MYC oncoprotein, and inducing rapid apoptosis in cancer cells. This cellular potency and mechanism of action translate to promising anti-cancer activity in human leukemia mouse xenograft models. Studies of leukemia cell line sensitivity identify mixed lineage leukemia (*MLL*) gene status and the level of B-cell lymphoma 2 (BCL2) family proteins as potential markers for selection of patients with greater sensitivity to fadraciclib. We show that the combination of fadraciclib with BCL2 inhibitors, including venetoclax, is synergistic in leukemic cell models, as predicted from simultaneous inhibition of MCL1 and BCL2 pro-survival pathways. Fadraciclib preclinical pharmacology data support its therapeutic potential in CDK9- or CDK2-dependent cancers and as a rational combination with BCL2 inhibitors in hematological malignancies. Fadraciclib is currently in Phase 1 clinical studies in patients with advanced solid tumors (NCT02552953) and also in combination with venetoclax in patients with relapsed or refractory chronic lymphocytic leukemia (CLL) (NCT03739554) and relapsed refractory acute myeloid leukemia (AML) or myelodysplastic syndrome (MDS) (NCT04017546).

Check for updates

OPEN ACCESS

Citation: Frame S, Saladino C, MacKay C, Atrash B, Sheldrake P, McDonald E, et al. (2020) Fadraciclib (CYC065), a novel CDK inhibitor, targets key pro-survival and oncogenic pathways in cancer. PLoS ONE 15(7): e0234103. https://doi.org/10.1371/journal.pone.0234103

Editor: Jed N. Lampe, University of Colorado Denver Skaggs School of Pharmacy and Pharmaceutical Sciences, UNITED STATES

Received: June 8, 2019

Accepted: May 19, 2020

Published: July 9, 2020

Copyright: © 2020 Frame et al. This is an open access article distributed under the terms of the Creative Commons Attribution License, which permits unrestricted use, distribution, and reproduction in any medium, provided the original author and source are credited.

Data Availability Statement: All relevant data are within the manuscript and its Supporting Information files. Original images from the Western blots are in: in DRYAD data repository DOI (doi:10.5061/dryad.44j0zpc9v).

Funding: This work was supported by Cyclacel Ltd in the form of salaries for SF, CS, CM, DB, and DZ. Part of the research was funded by Technology Strategy Board/Biomedical Catalyst (Project No: 101351). PW, EM, BA, PC and PS were funded by

CRUK Programme Grants (C309/A11566 and C2739/A22897) to the CRUK Cancer Therapeutics Unit at the ICR and also by sponsored funding from Cyclacel Ltd. The specific roles of these authors are articulated in the 'author contributions' section. The funders had no role in the study design, data collection and analysis, decision to publish, or preparation of the manuscript.

Competing interests: The updated Competing Interests are as follow:SF, CS, CM, DB and DZ are or have been employees and stakeholders of Cyclacel Ltd. PW, EM, BA, PS are or have been employees of The Institute of Cancer Research (ICR) which has a commercial interest in the discovery and development of CDK inhibitors and operates a rewards-to-inventors scheme. They have been involved in a commercial research collaboration with Cyclacel Limited and intellectual property arising from the program was licensed to Cyclacel. PW was a consultant/Scientific Advisory Board member to Cyclacel. PW was also involved in commercial research collaboration on CDK inhibitors with Merck KGaA and intellectual property was licensed to Merck KgA. PW was involved in commercial collaboration with Astex Pharmaceuticals and intellectual property was licensed to Astex. PW was a scientific founder, consultant, Scientific Advisory Board member and stock holder of Piramed Pharma (acquired by Roche) and intellectual property was licensed to Genentech. PW was a scientific founder, consultant, Scientific Advisory Board member, Board member and is a stockholder of Chroma Therapeutics and intellectual property was licensed to Chroma. PW is also a consultant/advisor to Astex Pharmaceuticals, CV6 Therapeutics and NextechInvest, and is a Non-Executive Director and stockholder of STORM Therapeutics. This does not alter our adherence to all the PLOS ONE policies on sharing data and materials.

Introduction

Cyclin-dependent kinases (CDKs) have been the subject of intense research for the last two decades because of their association with the cancer hallmarks of uncontrolled proliferation and increased survival. Following the successful development and approval of CDK4/6 inhibitors, the focus of pharmaceutical CDK inhibitor drug development in oncology has shifted to compounds regulating transcription of key oncogenic and pro-survival proteins [1].

Approximately twenty years ago it was recognized that transformed cells require continuous activity of RNA polymerase II (RNA pol II) to resist oncogene-induced apoptosis [2]. It was postulated that constitutive expression of one or more anti-apoptotic genes was essential to prevent oncogene-induced apoptosis and maintain the transformed state. In contrast non-transformed cells were relatively unaffected, at least in the short-term, by inhibition of transcription. This was the first demonstration that despite being an essential process, transcription may be a *bona fide* target in oncology with an acceptable therapeutic window. Two decades later, this theory is being evaluated through the development of more clinically applicable approaches to RNA pol II transcriptional inhibition, including small molecule drugs targeting CDK9, BRD4 and DOT1L methyltransferase [3–5].

First generation CDK inhibitors were found to inhibit both cell cycle and transcriptional CDKs to varying extents, including alvocidib (flavopiridol), seliciclib (CYC202) and dinaciclib. Several reports indicate that a predominant mechanism of action of these compounds is via RNA pol II transcriptional inhibition [1,2,6]. Importantly, inhibition of the CDK9 complex involved in transcriptional elongation was found to result in the loss of transcripts with short half-life. Much of the work to establish the mechanism of action of these agents has focused on the loss of MCL1, a pro-survival member of the BCL2 family [6]. It was demonstrated that: (i) MCL1 levels decrease rapidly upon treatment and precede apoptosis induction; (ii) depletion of MCL1 is sufficient to induce apoptosis, (iii) ectopic expression of physiological levels of MCL1 rescues cancer cells from transcriptional inhibitor compounds, and (iv) the pattern of sensitivity to transcriptional inhibitors across a panel of cancer cell lines closely mirrors the pattern of sensitivity to *MCL1* knockdown by siRNA. Thus, despite the coincident repression of many other transcripts, MCL1 loss is inextricably linked to the anti-cancer efficacy of this class of inhibitors.

In addition to the pharmacologic inhibition, selective CDK9 inhibition via siRNA has also resulted in MCL1 depletion and apoptosis induction [7].

MCL1 is one of the most frequently amplified genes in cancer [8]. In chronic lymphocytic leukemia (CLL) and multiple myeloma (MM), high levels of MCL1 are associated with the shortest progression free survival [9,10]. In acute myeloid leukemia (AML) and acute lymphoblastic leukemia (ALL), MCL1 is often elevated at relapse, suggesting a possible role for MCL1 in the survival of leukemia cells after chemotherapy [11]. Cells harboring amplifications surrounding the *MCL1* gene depend upon expression of this gene for survival [11]. Silencing *MCL1* (but not *BCL2L1* (*bcl-xL*)) induced cell death in a subset of triple negative breast cancer (TNBC) and non-small cell lung cancer (NSCLC) cell lines and was correlated with the expression of the various BCL2 family members [12–14]. Suppression of MCL1, but not BCL2 or BCL2L1 (other pro-survival members of the BCL2 family) triggered rapid apoptosis in CLL [15], MM [16] and AML, and cured AML-afflicted mice [17]. Importantly, AML cells were more sensitive to MCL1 depletion than non-transformed stem cells and myeloid progenitors, indicating a potential therapeutic window with appropriate dosing in patients.

Several studies have attempted to elucidate the relationship between MCL1 expression and specific genetic markers. For example, Kelly et al. [18] showed that MYC-driven mouse or human lymphomas were highly sensitive to depletion of MCL1, suggesting that evasion of apoptosis in MYC-overexpressing tumors may require upregulation of MCL1. Similar findings

have been reported for FMS related receptor tyrosine kinase (*FLT3)* internal tandem duplication (ITD) leukemia [19] and BCR-ABL driven chronic myeloid leukemia (CML) [20]. This may suggest a general mechanism whereby the survival of cells carrying strong oncogenic signals is maintained through overexpression of pro-survival MCL1 to counteract otherwise dominant pro-apoptotic triggers.

Decrease in MCL1 protein level has been demonstrated in peripheral blood samples from AML patients in response to treatment with dinaciclib [21]. However, the doses and schedules employed did not achieve durable suppression of MCL1 levels and were likely sub-optimal for induction of cell death, which could help to explain the absence of robust clinical responses. Indeed, primary AML samples treated ex-vivo with dinaciclib required 24 h exposure and downregulation of MCL1 to cause significant cell death [21]. Similarly, no decrease in MCL1 level was observed following 24 h infusion of alvocidib in patients with AML [22].

CDK2 is a key cell cycle regulator, with roles in phosphorylation and inactivation of the retinoblastoma protein (RB) tumor suppressor family and in controlling both G1/S and G2/M transitions [23]. CDK2 promotes DNA replication and plays a role in DNA damage response [24]. Tumors with overexpressed/amplified cyclin E are addicted to CDK2/cyclin E activity and highly sensitive to CDK2 inhibition [25,26]. Cyclin E/CDK2 phosphorylates and stabilizes MCL1 [27] suggesting that simultaneous inhibition of CDK9 and CDK2 will further facilitate MCL1 downregulation and apoptosis induction in tumors sensitive to this pathway.

There are two key success factors for CDK inhibitors that preferentially target CDK2 and CDK9. The first is a suitable pharmacokinetic profile, resulting in sustained target inhibition sufficient to trigger irreversible commitment to cell death, while avoiding prolonged exposure to normal cells. The second is identification of mechanistically sensitive tumors, which depend heavily on CDK9-dependent transcription of selected driver genes such as *MCL1*, *MYC*, *MYCN*, or which are reliant on dysregulated activation of CDK2/cyclin E. A good precedent for such patient selection is the identification of a specific breast cancer subset for CDK4/6 inhibitors [28].

Seliciclib, a first generation aminopurine inhibitor of CDK2, CDK7 and CDK9, has shown anti-proliferative activity in tumor cell lines and xenografts [29]. Patients treated with seliciclib have derived modest clinical benefit, as anti-cancer activity may have been compromised by low potency against the primary targets [30]. Moreover, analysis of pharmacodynamic markers and the pharmacokinetic profile of seliciclib in patients indicate that the rapid metabolism (short half-life) of seliciclib delivered orally may have resulted in transient rather than sustained modulation of the target, limiting commitment to cell death [31].

We sought to design a compound series based around the seliciclib scaffold, which is inherently CDK-selective, with the goal of improving metabolic stability and enhancing potency [32]. We previously reported a lead compound from this series, CCT068127, with reduced metabolism, enhanced selectivity towards CDK2 and CDK9 and superior therapeutic activity against human cancer cells [33]. Here we describe the synthesis and characterization of fadraciclib, the clinical candidate from this series. Fadraciclib is currently being evaluated in first-in-human studies in patients with advanced solid tumors (NCT02552953) and in combination with the BCL2 inhibitor, venetoclax, in patients with relapsed or refractory CLL (NCT03739554) and relapsed refractory AML or MDS (NCT04017546).

Materials and methods

Synthesis of fadraciclib, 2-pentanol, 3-[[6-[[(4,6-dimethyl-3-pyridinyl) methyl]amino]-9-(1-methylethyl)-9Hpurin-2-yl]amino]-, (2R,3S)

To a stirred solution of (4,6-dimethyl-pyridin-3-ylmethyl)-(2-fluoro-9-isopropyl-9H-purin-6-yl)-amine (300 mg 0.84mmol) in n-BuOH / DMSO (5 ml, 4:1) at room temperature under

an argon atmosphere, DIEA (1.7 ml, 10 eq, 8.4 mmol) was added followed by (2R,3S)-3-amino-pentan-2-ol (0.5g, 4.8 mmol). The flask was fitted with a condenser and the reaction mixture was placed in a preheated oil bath at 140˚C and stirred at this temperature for 72 h. The reaction mixture was allowed to cool to room temperature and the solvent was evaporated *in vacuo*. The residue was partitioned between ethyl acetate (50 ml) and water (50 ml), the aqueous phase was extracted with more EtOAc (2 x 25 ml) and combined organic phase was washed with brine (50 ml), dried ($MgSO_4$) and evaporated *in vacuo*. The residue was purified by flash gradient column chromatography on silica gel eluted with $CHCl_3$: MeOH (100: 0 →95: 5), to afford 55 mg of pure title compound. For further details on the synthesis of the series, refer to Wilson et al. [32].

NMR spectrum data: δ_H (250 MHz, $CDCl_3$) 095 (3, H, t J 7.5, $CHCH_2CH_3$), 1.06 (3 H, d, J 7.5, $CHCH_3OH$) 1.48 [6 H, d, J 7.5 $CH(C\underline{H}_3)_2$], 2.24 (3 H, s, CH_3), 2.4 (3 H, s, CH3), 3.92–3.82 (2 H, m, $NHCH_2$), 4.67–4.45 (3 H, m, $C\underline{H}EtC\underline{H}MeO\underline{H}$), 6.16 (1 H, s, br, NH), 6.87 (1 H, s ArH), 7.37 (1 H, ArH), 8.31 (1 H, s, ArH); δ_C (250 MHz, $CDCl_3$) 160.11 (C), 157.68 (C), 154.57 (C), 149.42 (CH), 146.38 (C), 134.54 (CH), 129.24 (C), 124.84 (CH), 71.52 (CH), 59.65 (CH), 40.33 (CH_2), 24.94 (CH_2), 23.89 (CH_3), 23.52 (2 x CH_3), 1737 (CH_3), 12.57 (CH_3); m/z 398 (M + H)

Determination of kinase inhibitory potency and selectivity *in vitro* using recombinant enzymes

Kinase assays for CDK2, CDK4, CDK7 and CDK9 were carried out as described previously using recombinant CDK/cyclins generated at Cyclacel Ltd, Dundee, UK [29,34,35].

Profiling of 1 μM fadraciclib against a 256-kinase selectivity panel was performed at Carna Biosciences. Enzymes that inhibited >50% at the tested concentration were selected for IC_{50} determination. IC_{50} values were determined from a 10-point concentration curve using ATP concentrations approximating K_m values. The value obtained for the reaction control (complete reaction mixture) was set as 0% inhibition and for the background (no enzyme) as 100% inhibition. The percentage inhibition of each test solution was calculated, which allowed the IC_{50} value to be determined from concentration versus percentage inhibition curves by fitting to a four parameter logistic curve.

Mining publicly available data for the dependence of cancer cell lines on CDKs inhibited by fadraciclib

We analyzed data available from the Cancer Dependency Map to determine the effects of CRISPR-Cas9 knockdown of CDKs inhibited by fadraciclib on the viability of >700 cancer cell lines, including those used in the present study. Results were shown as a CERES gene effect that scores the Dependency of cell lines on a given gene Score ((https://depmap.org/portal/).

Cell lines and tissue culture conditions

The work presented in this report was performed on a panel of human cancer cell lines, including Colo205, TNBC, AML and several cell lines of non-malignant origin. A list of the cell lines used in the project, along with a brief description and culture conditions, is presented in S1 Table.

Colo205 was derived from a colorectal carcinoma and obtained from ECACC. The breast cell line panel consisted of lines derived from two TNBC tumors and a *HER2*-amplified breast tumor. In addition, two immortalized cell lines were derived from healthy, non-malignant

breast tissue. All breast panel cell lines were obtained from ATCC, with the exception of Cal51 which was purchased from DSMZ.

Seven of the thirteen AML cell lines contain *MLL* rearrangements (*MLL*r) or partial tandem duplications (PTD): EOL-1 (*MLL*-PTD), ML-2 (*MLL-AF6*), MOLM-13 (*MLL-AF9*), MV4-11 (*MLL-AF4*), NOMO-1 (*MLL-AF9*), OCI-AML2 (*MLL*-PTD), THP-1 (*MLL-AF9*) and 6 contain no *MLL* abnormalities: HEL, HL60, Kasumi-1, KG-1, OCI-AML5 and PL-2. All AML cell lines were purchased from DSMZ with the exception of THP-1 and HL-60, which were sourced from ECACC and ATCC, respectively.

All cell lines were grown in tissue culture medium recommended by the supplier in incubators at 37˚C and 5% CO_2. Information on the cell lines including their classification, origin, supplier, doubling time, tissue culture media, *MLL* gene status and other molecular and genetic characteristics are summarized in S1 Table.

Determination of cell viability using the resazurin reduction assay

Solid tumor cell lines were seeded in 100 μl medium into 96-well plates at an appropriate cell number determined by a cell dilution assay (Cal51–2000 cells/well; Colo205, MCF10A and 184A1–3000 cells/well and MDA-MB-468, HCC1954–4000 cells/well). AML suspension cells were seeded in 96-well plates at approximately 3000 cells per well. Cells were incubated overnight at 37˚C, 5% CO_2. The following day, 100 μl fresh medium containing increasing concentrations of appropriate compound was added and the cells were incubated for up to 72 h at 37˚C, 5% CO_2. For pulse treatment of solid (adherent) tumor cell lines, medium was aspirated and replaced with fresh drug-free medium. For pulse treatment of suspension lines, plates were centrifuged at 478 xg for 5 min at the end of the incubation period with compound, medium containing compound was carefully aspirated and replaced with fresh compound-free medium. A total of 72 h after the start of treatment, 20 μl of a 10X stock of resazurin sodium salt (Sigma-Aldrich, Dorset, UK) prepared in medium was added to the cells and incubated for up to 3 h. Absorbance was measured on the Wallac Victor plate reader (Cambridge, UK) at 544–595 nm. $IC_{50/70/90}$ calculations were determined in XLFit.

Cell viability analysis by flow cytometry

AML cells were seeded at 5 x 10^5 cells/ml in a 6-well plate and incubated with the test compound or vehicle control (DMSO) for 6, 8 or 10 h. At the end of the incubation period, cells were collected into Falcon tubes, pelleted via centrifugation (5 min at 478 x g), and medium containing compound was aspirated. The cells were resuspended in 4 ml of fresh compound-free medium and returned to the 6-well plates for further incubation. Following compound addition, 100 μl (at 24 h), 75 μl (at 48 h), 50 μl (at 72 h) or 30 μl (at 96 h) of a homogeneous suspension of cells were removed from the culture, made up to 100 μl with medium and mixed with 100 μl Guava Viacount reagent (Millipore, Nottingham, UK) in a 96-well plate. Samples were incubated for 20 min in the dark, mixed and then analyzed by GuavaSoft 2.6 software on the Guava Easycyte 8HT (Millipore). Guava Viacount software was used to gate and calculate the proportion of viable and dead cells in the population.

Cell cycle analysis by flow cytometry

Colo205 cells were seeded at 8 x 10^5 cells per 10 cm dish in a total of 9 ml medium and incubated overnight at 37˚C, 5% CO_2. The following day 1 ml of medium containing 10x test agent or vehicle was added to the cells, which were then incubated for 24 h at 37˚C, 5% CO_2. Cells were collected, prepared, propidium iodide stained for DNA content analysis, and analyzed as

described previously [29]. Analysis was performed using BD Cell Quest 3.3 software on a BD LSR flow cytometer.

TNBC cell lines were seeded at 1 million cells per 10 cm dish in a total of 9 ml medium and incubated overnight at 37˚C, 5% CO_2. The following day 1 ml of 10x stock of fadraciclib or fresh medium were added to the cells for an 8 h pulse before the medium was removed and replaced with fresh compound-free medium. Cells were incubated for a further 16 h at 37˚C, 5% CO_2 before samples were collected in 50 ml Falcon tubes (including all cells in suspension in original medium, cells collected from the PBS wash and adherent cells collected from trypsinisation). The pooled collected cells were washed once with PBS, followed by fixation in ice-cold 70% ethanol overnight at -20˚C. 5 x 10^5 cells, as recommended by the cell cycle kit instructions, were washed once in 1 ml PBS, centrifuged at 200 x g for 5 min then resuspended in PI solution from Guava cell cycle reagent kit from Millipore (4500–0220) for 30 min before analysis by GuavaSoft 2.6 software on a Guava Easycyte 8HT.

AML cells were seeded in a 6-well plate at 5 x 10^5 cells/ml and incubated with test compound for 16 h. Cells were collected and washed once with PBS, followed by fixation in ice-cold 70% ethanol overnight at -20˚C. Samples were prepared for propidium iodide staining of DNA to determine the proportion of cells in different cell cycle phases within the population as per the Guava Cell Cycle Reagent manufacturer's instructions. Briefly, cells were washed once in PBS and then resuspended in 100 μl PBS. An equivalent volume of Guava Cell Cycle Reagent was added to the cells and incubated for 20 min in the dark. The stained cell suspension was then resuspended and analyzed by GuavaSoft 2.6 software on a Guava Easycyte 8HT.

Quantitative Real-Time (RT) PCR analysis

AML cells were seeded at 5 x 10^5 cells/ml in appropriate medium and treated for up to 3 hours with various concentrations of fadraciclib or vehicle control (DMSO). Cells were collected every hour, washed once in PBS, pelleted by centrifugation and pellets were snap-frozen in liquid nitrogen and stored at -20˚C. Total RNA was extracted using the RNAeasy minikit (Qiagen, Manchester, UK) according to the manufacturer's instructions. Quantitative real-time PCR was carried out on the Lightcycler II (Roche, Basel, Switzerland) using the One-Step RT-PCR SYBRGreen kit as recommended by the manufacturer and using validated gene-specific primers from Qiagen. The cycling conditions were 50˚C for 20 min for the RT step, then 95˚C for 15 min and 40 cycles of 94˚C for 15 s, 55˚C for 20 seconds and 72˚C for 20 s. Beta-2 microglobulin (B2M) was used as a housekeeping control for normalization of the data. Each sample was tested in duplicate. Fold changes in gene expression were calculated and normalized to B2M expression.

Western blot analysis

Protein samples for Western blot analysis were prepared as follows. AML cells were seeded at 5 x 10^5 cells/ml and solid tumor cell lines were seeded at approximately 1 million cells per 10cm dish in the appropriate medium and incubated overnight. Cells were treated for up to 24 h with various concentrations of fadraciclib or DMSO as vehicle control. Cells were collected at various time-points as indicated and centrifuged for 5 min at 478 x g. Medium was removed and cells were washed once in PBS. Cells were pelleted by centrifugation and the pellets were snap-frozen in liquid nitrogen and stored at -20˚C until analysis. Pellets were lysed in NP40 cell lysis buffer (Life Technologies, Paisley, UK), PMSF, sodium beta-glycerophosphate and protease inhibitor cocktail (1:1000, Sigma-Aldrich) and sonicated. The soluble protein fraction was obtained after centrifugation at 14,000 x g for 10 min. The protein yield was determined

using the BCA protein determination kit (Pierce, Thermo Fisher Scientific, Northumberland, UK) as per the manufacturer's instructions.

Total protein (25μg) was mixed with 4x gel loading buffer and 1x reducing agent and separated in precast NuPAGE 10%, 4–12% Bis-Tris or 3–8% Tris-Acetate polyacrylamide gels using denaturing electrophoretic conditions (Life Technologies). Proteins were transferred to nitrocellulose membranes using an IBlot transfer unit (Life Technologies). Membranes were blocked in 5% nonfat milk in PBS with 0.05% Tween 20 (PBST) for 2 hours and incubated overnight at 4˚C with primary antibody diluted in 5% milk in PBST, or 3% BSA for phospho-specific antibodies. Membranes were washed 3 x 10 min in PBST and incubated for 1 h in 5% milk in PBST containing horseradish peroxidase-conjugated secondary antibody. Membranes were washed as before and incubated with enhanced chemiluminescence solution and exposed to X-ray film (Amersham, VWR, Leicestershire, UK). Protein bands were quantified using ImageJ software. Alternatively, Clarity ECL solution was used and images were captured and analyzed on the Chemidoc (both Biorad, UK). The antibodies and their final concentrations used are listed in S2 Table.

In vitro combination studies of fadraciclib with BCL2 inhibitors

Venetoclax (ABT-199), ABT-263 and ABT-737 were purchased from Selleck Chemicals. To assess potential synergistic interactions, the concomitant treatment regimen involved simultaneous treatment of cells for 72 h, alongside suitable controls of cells treated with the individual compounds alone for 72 h. At the end of the treatment period, cell viability was assessed using the Alamar Blue Cytotoxicity Assay (see above). Combination Index (CI) values were calculated using the method of Chou and Talalay [36]. A combination index (CI) value of 0.9–1.1 indicated an additive drug interaction, a CI value of >1.1 was considered antagonistic and CI <0.9 was considered synergistic.

Assessment of *in vivo* antitumor activity

EOL-1 human eosinophilic leukemia xenograft model. Female athymic CB-17 SCID (Harlan Sprague-Dawley) mice between 6–7 weeks of age weighing approximately 22 grams were housed in a 12-h light-dark cycle at 70–74˚F (21–23˚C) and 40–60% humidity. Animals were fed water *ad libitum* (reverse osmosis, 2 ppm Cl2) and an irradiated standard rodent diet (Teklad 2919) consisting of 19% protein, 9% fat, and 4% fiber. Mice were injected subcutaneously into the right flank with $1x10^7$ EOL-1 cells in 100 μl of a 1:1 HBSS:Matrigel solution. When tumors reached approximately 125–250 mm^3, animals were matched by tumor volume and then randomized into treatment and control groups before dosing was initiated. Fadraciclib was formulated weekly in sterile ddH_2O and dosed by oral gavage once a day on a 5 days on/2 days off schedule per cycle for 2 cycles. Cytarabine, also known as cytosine arabinoside (AraC), was administered by daily ip injection for 5 days only. Agent toxicity was assessed by effects on body weight and clinical signs. Animals were weighed twice weekly using a digital scale (Ohaus SP601). Beginning on Day 0, tumor dimensions were measured twice weekly by digital caliper (Fowler Ultra-Cal IV); tumor volume was calculated using the formula $TV = width^2 \times length \times 0.52$. At study completion, percent tumor growth inhibition (%TGI) values were calculated and reported for each treatment group (T) versus control (C) using initial (i) and final (f) tumor measurements by the formula %TGI = 1- Tf-Ti / Cf-Ci. Statistical differences in tumor volume were determined using a two-tailed One-Way Analysis of Variance (ANOVA) followed by the Dunnett's multiple comparisons test comparing treated groups with control. For animal welfare the following methods were used: (a) methods of sacrifice: isoflurane anesthesia followed by cervical dislocation; (b) methods of anesthesia and/or

analgesia: isoflurane anesthesia and (c) efforts to alleviate suffering: carprofen if animal veterinarian decides necessary or euthanasia.

HL-60 human promyelocytic leukemia xenograft model. Five-week-old female athymic NCr-*nu/nu* mice were purchased from Harlan Sprague-Dawley (Prattville, AL). The animals were housed in microisolator cages, with up to five animals per cage in a 12-h light/dark cycle. The animals received filtered water and sterilized rodent diet (Harlan-Teklad TD8656) *ad libitum*. Each mouse was implanted subcutaneously near the right flank with ten million HL-60 cells using a 23-gauge needle and allowed to grow. Tumors were allowed to reach 75–144 mm^3 in size before treatment initiation. Fadraciclib was dosed by oral gavage daily on a 5 days on/2 days off schedule per cycle for 2 cycles (Q1Dx5/2). The %TGI values were calculated and statistical analyses performed as above.

Results

Synthesis and characterization of fadraciclib

We disclose for the first time (Fig 1A) the chemical structure of fadraciclib, a second generation clinical candidate based on the aminopurine scaffold of the CDK inhibitor seliciclib. Seliciclib is an ATP competitive inhibitor of CDK2, CDK7 and CDK9, with an average anti-proliferative activity (IC_{50}) in tumor cells of around 15 μM [29]. Seliciclib has been studied extensively in preclinical and clinical settings in both oncology and non-malignant, proliferative and autoimmune conditions NCT00999401, ISRCTN36667085, NCT02160730 and NCT02649751) [30]. It has moderate aqueous solubility and is readily absorbed when administered orally. However, seliciclib is rapidly converted to a less potent carboxylic acid analogue through first pass oxidative metabolism [37]. Medicinal chemistry efforts to increase target CDK inhibitory potency and enhance the metabolic stability of seliciclib resulted in the synthesis of 6-pyridylmethylaminopurines with increased aqueous solubility, metabolic stability and significantly improved target inhibition and anti-proliferative activity [32,33].

The clinical development candidate selected from this series is fadraciclib. The synthetic procedure and spectral data are described in Materials and Methods. Fig 1A shows the chemical structure of fadraciclib together with seliciclib and the previously described lead compound CCT068127 [33].

To confirm retention of the desired CDK selectivity, and select the most promising compounds from this series, analogues were screened for inhibition of a panel of CDK enzymes encompassing the principal targets of seliciclib, CDK2, CDK7 and CDK9, and counter-screened against CDK4. The CDK selectivity profiles of seliciclib and the purine analogues are similar, but not identical (Fig 1B). Seliciclib inhibits CDK2, CDK7, and CDK9, in that order, whereas fadraciclib and CCT068127 are more selective for CDK2 and CDK9 and inhibit CDK7 to a much lesser extent. All three compounds exhibit low potency against CDK4. IC_{50} values for fadraciclib against CDK2 and CDK9 are approximately 20-fold lower than those for seliciclib, which is likely to result in improved cellular anti-proliferative activity *in vitro*.

To further characterize fadraciclib, its potency was investigated in Colo205 human colon cancer cells in comparison to seliciclib, CCT068127 and alvocidib. The IC_{50} value of 13.3 μM for seliciclib in Colo205 (S3 Table) was in agreement with the reported average anti-proliferative IC_{50} for this compound [25]. In contrast, the IC_{50} values for the two most potent analogues, fadraciclib and CCT068127, were 0.31 μM and 0.82 μM, respectively, which is comparable to alvocidib (0.129 μM S4 Table). The increased potency of fadraciclib was confirmed in a human tumor cell line panel (S5 Table), in which it exhibited an approximately 34-fold enhanced anti-proliferative activity compared with seliciclib.

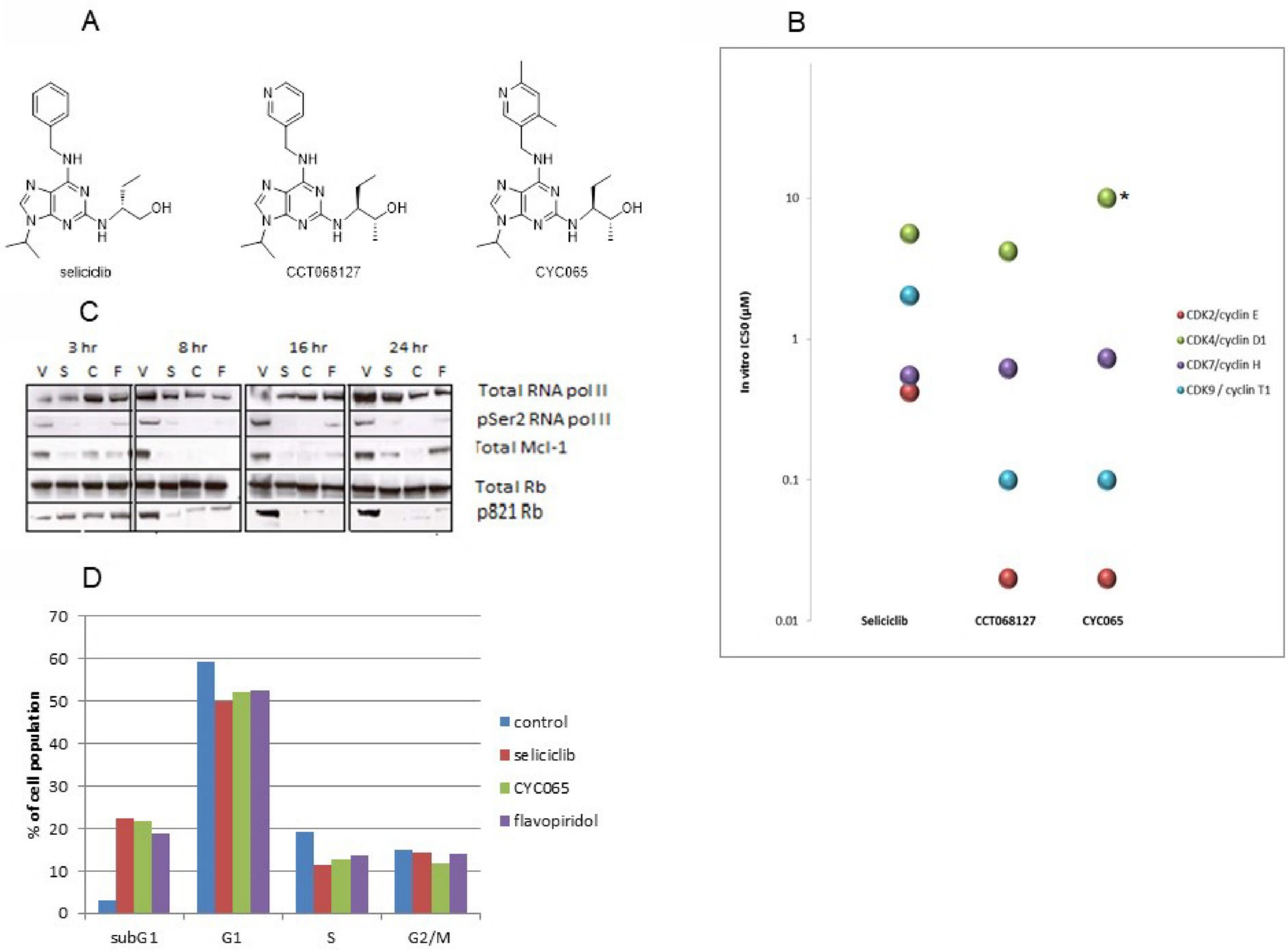

Fig 1. Enhanced potency and conserved mechanism of action of CCT068127 and fadraciclib (CYC065) over seliciclib. A. Chemical structures of seliciclib, and the 6-pyridylmethylaminopurine derivatives, CCT068127 and the clinical candidate, fadraciclib (CYC065). **B.** CDK selectivity profile of seliciclib, CCT068127 and fadraciclib (CYC065). * $IC_{50} > 10$ μM. **C.** Confirmation of mechanism of action of the derivative compounds. Colo205 colon cancer cells were treated with vehicle (V), seliciclib (S) (26.6 μM), fadraciclib (CYC065) (C) (0.62 μM) or alvocidib (flavopiridol) (F) (0.26 μM) at equivalent of 2 x IC_{50} for up to 24 h. Cells were harvested at the indicated time-points and the expression levels of RNA pol II, RNA pol II CTD pSer2, RB and MCL1 were examined by Western blotting. D. Colo205 cells were treated for 24 h with 2 x IC_{50} seliciclib, fadraciclib (CYC065), alvocidib (flavopiridol) or vehicle control, then harvested and fixed for propidium iodide staining to examine the cell cycle profile.

https://doi.org/10.1371/journal.pone.0234103.g001

The cellular mechanism of action of fadraciclib was confirmed in Colo205 cells by examining the impact of treatment for up to 24 h by Western blotting (Fig 1C) and flow cytometry (Fig 1D). In agreement with the observed changes in key CDK targets after seliciclib treatment, fadraciclib treatment resulted in a rapid and robust decrease in RNA pol II C-terminal domain (CTD) pSer2 (a target of CDK9), loss of MCL1 within 3 h, and a decrease in RB pThr821 (a potential CDK2 site) within 8 h. Interestingly, the effect of fadraciclib on these targets were sustained up to 24 h. In contrast, there was evidence of recovery in the levels of RNA pol II CTD pSer2 and particularly MCL1, despite the continued presence of seliciclib or alvocidib (previously reported in [38]).

Together with seliciclib and alvocidib for comparison, the cellular consequences of fadraciclib was examined by flow cytometry in Colo205 cells (Fig 1D).The results revealed the

predominant cellular effect to be induction of cell death, as measured by an increase in the sub-G1 population. The robust increase in percentage of cells in sub-G1 made it difficult to draw conclusions on any specific cell cycle perturbations induced by the compounds, although there was evidence of a decrease in cells in both G1 and S phases. The absence of a marked cell cycle arrest is in agreement with the published literature for this class of compounds [33] indicating that the predominant mechanism of action (at concentrations equivalent to 2 x IC_{50}) is related to inhibition of CDKs regulating transcription resulting in induction of apoptosis.

In summary, fadraciclib is significantly more potent against CDK2 and CDK9 and has enhanced effects compared to seliciclib on cell growth and induction of cell death. Fadraciclib shows a more sustained effect on MCL1 in comparison with seliciclib or alvocidib, resulting in its selection as the preferred clinical candidate.

Kinome profiling

Identifying compounds with the appropriate CDK inhibitory profile to maximize effects on transcription, cell cycle and cancer cell death, while retaining sufficient selectivity to avoid unwanted collateral toxicities, is challenging. Due to its essential nature, inhibition of CDK1 appears not to be desirable [39].

To more fully characterize the kinase selectivity of fadraciclib, *in vitro* screening was performed with 1 μM fadraciclib against a panel of 256 kinases (Carna Biosciences). At this concentration, fadraciclib inhibited only 9 kinases >50%, consisting of 7 CDK enzymes, and 2 CDK-like kinases (Fig 2 and S6 Table). IC_{50} values for fadraciclib against each of these enzymes were determined at ATP concentrations close to the respective K_m values Fadraciclib was shown to be most potent against CDK2/cyclin A (IC_{50} = 5 nM), followed by CDK5/p25, CDK9/cyclin T1 and CDK3/cyclin E1 (IC_{50} values of 21, 26 and 29 nM, respectively) (Table 1). Fadraciclib is more than 40-fold less potent against CDK7/cyclin H, CDK4/cyclin D3 and CLK2 and more than 100-fold less potent against CLK1 and CDK1/cyclin B (Table 1). Like seliciclib, fadraciclib is extremely selective for CDK over non-CDK enzymes.

To determine the potential contribution of inhibiting CDK3 and CDK5, as compared to CDK2 and CDK9, we analyzed data publicly available from genome-wide CRISPR-Cas9 screens in >700 cancer cell lines (from the Cancer Dependency Map project). The results show that in almost all cancer cell lines tested, including the cell lines described in S5 Table, genetic loss of CDK3 and CDK5 has a minimal impact on cell viability. This contrasts with dependency of cancer cell lines revealed following the genetic loss of CDK2 and particularly CDK9 (S6 Fig). Thus the predominant pharmacological effect of fadraciclib on cancer cells is likely to be mediated by inhibition of CDK2 and CDK9.

Characterization of fadraciclib mechanism of action in target cancer and non-transformed cell lines

Treatment strategies for TNBC represents an unmet medical need, as it is one of the most highly proliferative and aggressive types of breast cancer with a poor prognosis [40]. CDK2/9 inhibitors appear to be promising treatments for TNBC for several reasons. CDK2/cyclin E activity is elevated in a significant proportion of TNBC [41]. *MCL1* is commonly amplified and correlates with a poor prognosis [9]. TNBC tumors express high levels of the *MYC* oncogene product compared to other breast cancer subtypes [42]. These features may contribute to the aggressive and refractory nature of TNBC tumors. The properties of fadraciclib, including inhibition of CDK2 activity and depletion of transcripts with a short half-life, such as *MCL1* and *MYC*, as a result of inhibition of CDK9 [43] provide a strong therapeutic rationale to test this agent in TNBC models.

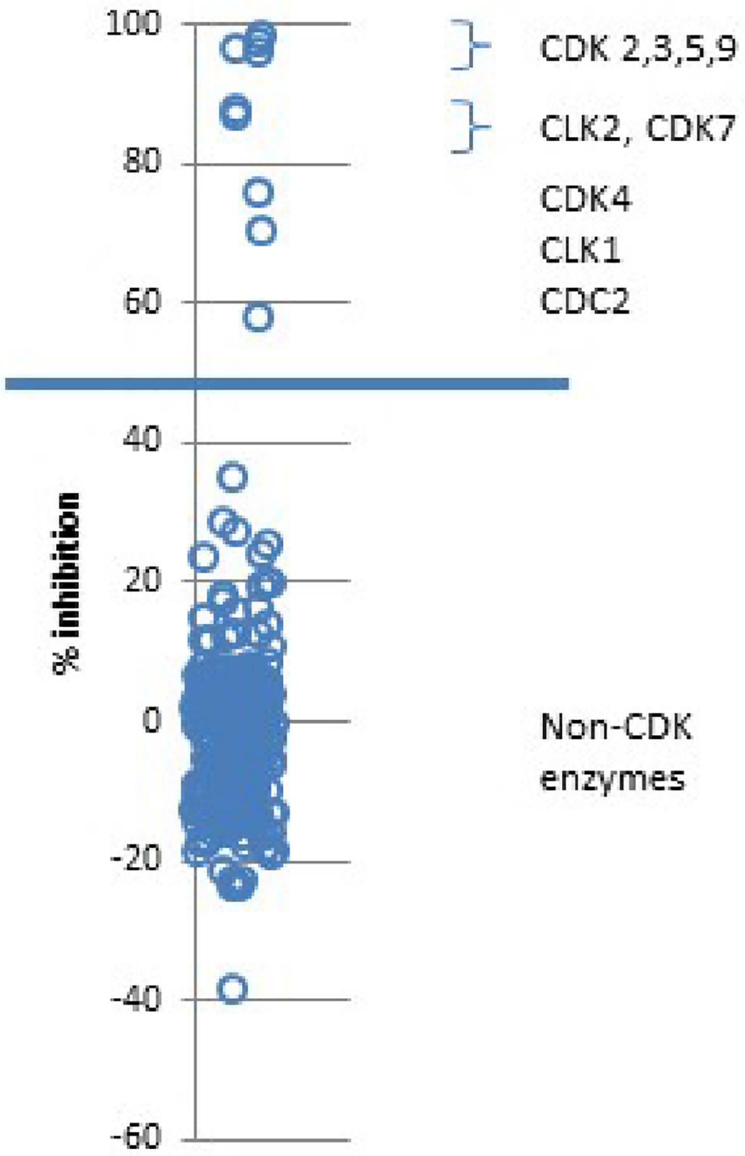

Fig 2. *In vitro* CDK selectivity of fadraciclib (CYC065). Fadraciclib (CYC065) (1 μM) was profiled against a 256 kinase selectivity panel. The value obtained for the reaction control (complete reaction mixture) was set at 0% inhibition, and the value of the background (no enzyme) was set as 100% inhibition. Percentage inhibition of each enzyme by fadraciclib (CYC065) is indicated in the graph. Nine CDK and CDK-like enzymes were inhibited greater than 50%.

https://doi.org/10.1371/journal.pone.0234103.g002

Throughout the study of CDK9 targeting transcriptional inhibitors (such as CCT068127, alvocidib, dinaciclib) it is clear that the onset of biochemical events and subsequent induction of apoptosis are rapid [29,34,44]. Within approximately 30 minutes, RNA pol II CTD pSer2 levels are diminished, followed by loss of MCL1 within a few hours, triggering an irreversible commitment to cell death [29,34,44], This suggests that while clinical exposure of several hours may be required to commit cells to apoptotic death, more prolonged target inhibition may not be necessary.

Table 1. CDK inhibitory potency and selectivity of fadraciclib (CYC065). IC_{50} values determined from a 9-point concentration curve at around K_m [ATP] (Carna Biosciences). The most potently inhibited kinases (IC_{50} < 30nM) are highlighted in bold.

Kinase	IC_{50} ± SD (nM)	Fold difference relative to CDK2
**CDK2/cyclin A**	**4.5 ± 0.4**	**1.0**
**CDK5/p25**	**20.5 ± 1.0**	**4.6**
**CDK9/cyclin T1**	**26.2 ± 2.0**	**5.8**
**CDK3/cyclin E1**	**28.9 ± 3.0**	**6.4**
CDK7/cyclin H1/MAT1	193 ± 20	42.9
CDK4/cyclin D3	232 ± 27	51.6
CLK2	252 ± 8	56.0
CLK1	549 ± 81	122.0
CDK1/cyclin B1	578 ± 41	128.4

https://doi.org/10.1371/journal.pone.0234103.t001

Indiscriminate inhibition of proliferation for prolonged periods is likely to be universally toxic to both malignant and healthy tissues, leading to significant gastrointestinal and bone marrow toxicity, regardless of CDK-specificity [45]. For example, in the Phase 1 trial assessing the safety and efficacy of a 72 h infusion schedule of alvocidib, toxicities were deemed to be unacceptable and the schedule was not efficacious [46,47]. These observations provided the impetus for utilizing clinically relevant durations and exposures of fadraciclib in preclinical studies, as guided by preclinical animal pharmacokinetics and toxicology, to minimize toxicity to healthy tissues.

We therefore examined the efficacy and cellular mechanism of action of fadraciclib in a small panel of breast cancer cell lines, including two human TNBC cell lines (Cal51 and MDA-MB-468) and the *HER2*-amplified HCC1954 breast cancer cell line. Non-transformed MCF10A and 184A1 cell lines were also included to assess differences in cellular response between cancer and non-transformed cell lines, which may have implications for the potential therapeutic window. Since we had observed complete loss of RNA pol II CTD pSer2, MCL1 and RB pThr821 by 8 h in Colo205 cells, an 8 h pulse treatment was selected and compared with cellular cytotoxicity of a continuous 72 h treatment in the breast panel (Table 2). The IC_{50} value established from a standard 72 h viability assay was determined to be ≤0.4 μM in all five cell lines. After 8 h pulse treatment with fadraciclib, the cellular IC_{50} value remained in the submicromolar range in all three breast cancer cell lines. In contrast, non-transformed MCF10A and 184A1 cells were much less sensitive to pulse treatment (IC_{50} = 3.9 μM), revealing a difference in sensitivity between the cancer and non-transformed models tested using the pulse dosing schedule.

Unsynchronized cell lines were treated with up to an 8 h pulse of 0.5 or 1 μM fadraciclib. Levels of key proteins related to CDK9 inhibition were determined (Fig 3A) together with biochemical evidence of cell death (Fig 3B). Reduced levels of RNA pol II CTD pSer2 were observed by 4–8 h fadraciclib treatment in all cell lines tested (Fig 3A). Consequently, there was also a clear reduction in the levels of both MCL1 and MYC protein in all cell lines. Importantly, PARP cleavage, indicating apoptotic induction, was only observed in the three breast cancer cell lines and not in non-transformed MCF10A cell line (Fig 3A). This demonstrates that although upstream effects of the compound are similar in cancer and non-transformed cell lines, fadraciclib only induces apoptosis in cancer cell lines at the concentrations used. This may reflect a higher dependency on MCL1 and MYC, or apoptotic priming, in cancer cell lines.

Table 2. Short pulse treatment with fadraciclib (CYC065) reveals a therapeutic window *in vitro*.

Cell Line	Classification	Fadraciclib 8 h pulse IC_{50} ± SD (μM)	Fadraciclib continuous IC_{50} ± SD (μM)
Cal51	TNBC	0.68 ± 0.20	0.23 ± 0.04
MDA-MB-468	TNBC	0.99 ± 0.34	0.38 ± 0.17
HCC1954	HER2 amplified	0.48 ± 0.05	0.40 ± 0.03
MCF10A	Non-malignant	3.89 ± 1.04	0.40 ± 0.03
184A1	Non-malignant	3.87 ± 2.10	0.25 ± 0.01

https://doi.org/10.1371/journal.pone.0234103.t002

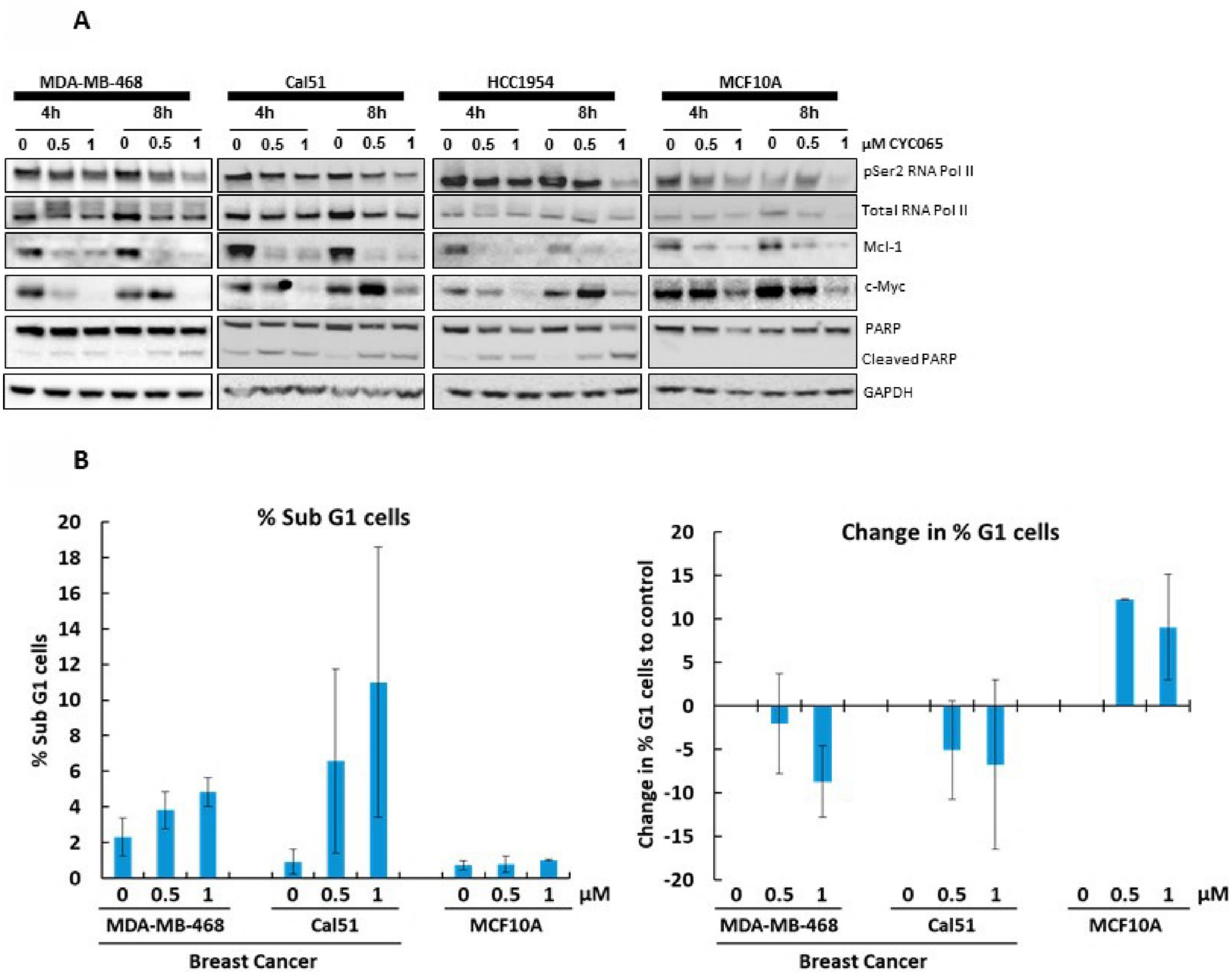

Fig 3. Fadraciclib (CYC065) is selectively cytotoxic to breast cancer cells. A. Representative human breast cancer cell lines and non-transformed cells derived from healthy breast tissue were exposed to fadraciclib (CYC065) for up to 8 h, and the expression levels of selected proteins was examined by Western blotting. **B**. Cells were treated with vehicle, 0.5 μM or 1.0 μM fadraciclib (CYC065) for an 8 h pulse, then cells were harvested at 24 h for cell cycle analysis by propidium iodide staining. Error bars denote SD.

https://doi.org/10.1371/journal.pone.0234103.g003

To further understand the difference in fadraciclib sensitivity between breast cancer and non-transformed cell lines, flow cytometry profiles were analyzed after an 8 h pulse of fadraciclib followed by incubation in drug-free medium for a further 16 h (Fig 3B). Consistent with the accumulation of cleaved PARP by Western blotting exclusively in cancer cell lines, induction of a sub-G1 population, indicative of cell death, was observed only in the cancer cell lines. In contrast, the non-transformed cell line MCF10A displayed an increase in the G1 population (Fig 3B), potentially indicative of cell cycle arrest. The differential sensitivity of cancer cells to apoptosis, together with the use of the pulse schedule, suggests a promising therapeutic window for fadraciclib, with implications for future clinical development.

Sensitivity of AML, a MCL1-dependent disease, to fadraciclib

MCL1 has been shown to be critical for survival of AML cells across multiple genetic subtypes, including the *MLL*r and *FLT3*-ITD subtypes, which are associated with a poor prognosis [48,49]. Because of the direct involvement of CDK9/cyclin T in MLL-driven transcription, it is predicted that CDK9 inhibition may have a significant impact in *MLL*-rearranged disease. We therefore selected a panel of human AML cell lines, ensuring adequate representation of *MLL*r and *FLT3*-ITD subtypes, to examine the activity of fadraciclib in AML.

Seven of the cell lines carry *MLL* gene rearrangements (www.dsmz.de/catalogues/catalogue-human-and-animal-cell-lines.html), including translocation with different partners such as *AF4* (MV4-11), *AF6* (ML-2) and *AF9* (MOLM-13, Nomo-1, THP-1), and partial tandem duplications (EOL-1 and OCI-AML2). In addition, two cell lines (MOLM-13 and PL21) carry *FLT3*-ITD, one has a *FLT3* mutation (MV4-11) and another amplified *MYC* (HL60).

As a first step, several of the AML cell lines were used to establish the minimum duration of pulse treatment to achieve optimal cellular response. As an example, Kasumi-1 cells were treated with 0.5 μM or 1.0 μM fadraciclib for up to 6 h and effects on levels of MCL1 protein and cleaved PARP were determined every hour (S1A Fig). MCL1 levels were strongly suppressed at 1 h and further depleted as treatment continued. Cleaved PARP was detectable at 2 h and reached a plateau at approximately 5–6 h following compound addition, suggesting that MCL1 depletion preceded the induction of apoptosis rather than being a consequence of it. A Viacount assay was employed to determine the proportion of dead cells and confirmed that a 6 h pulse treatment was sufficient to induce apoptosis in up to 80% of the cells (S1B Fig). Effect on viability of 6 h-, 8 h- and 10 h-pulses was also compared using the resazurin reduction assay with 72 h continuous treatment in AML cell lines. No significant differences in $IC_{50/70/90}$ values were observed between pulse and prolonged continuous treatments (data not shown). Based on these results, a 6 h pulse of exposure was selected for further characterization of AML cell line sensitivity to fadraciclib.

Asynchronously growing AML cells were treated with increasing concentrations of fadraciclib for 6 h and then maintained for an additional 66 h in compound-free medium. Cell viability was assessed using the resazurin reduction cytotoxicity assay and the $IC_{50/70/90}$ values determined (Fig 4A). The majority of AML cell lines were highly sensitive to fadraciclib treatment. Six hour incubation in low micromolar or sub-micromolar concentrations of fadraciclib resulted in 90% inhibition of cellular proliferation and yielded IC_{50} values very similar to 72 h continuous treatment (9 out of 13 cell lines; S7 Table). On average, *MLL*r/*MLL*-PTD AML cell lines tested were more sensitive to fadraciclib than AML cell lines without *MLL*r/*MLL*-PTD ($IC_{50/70/90}$ values; p = 0.048, 0.035 and 0.03, respectively; see inset Fig 4A). The only exception in the *MLL*r/*MLL*-PTD subset was the THP-1 cell line which was less sensitive than all other *MLL*r/*MLL*-PTD cell lines. Interestingly, a similar correlation between *MLL* status and sensitivity to fadraciclib was observed in a panel of ALL cell lines (S2 Fig).

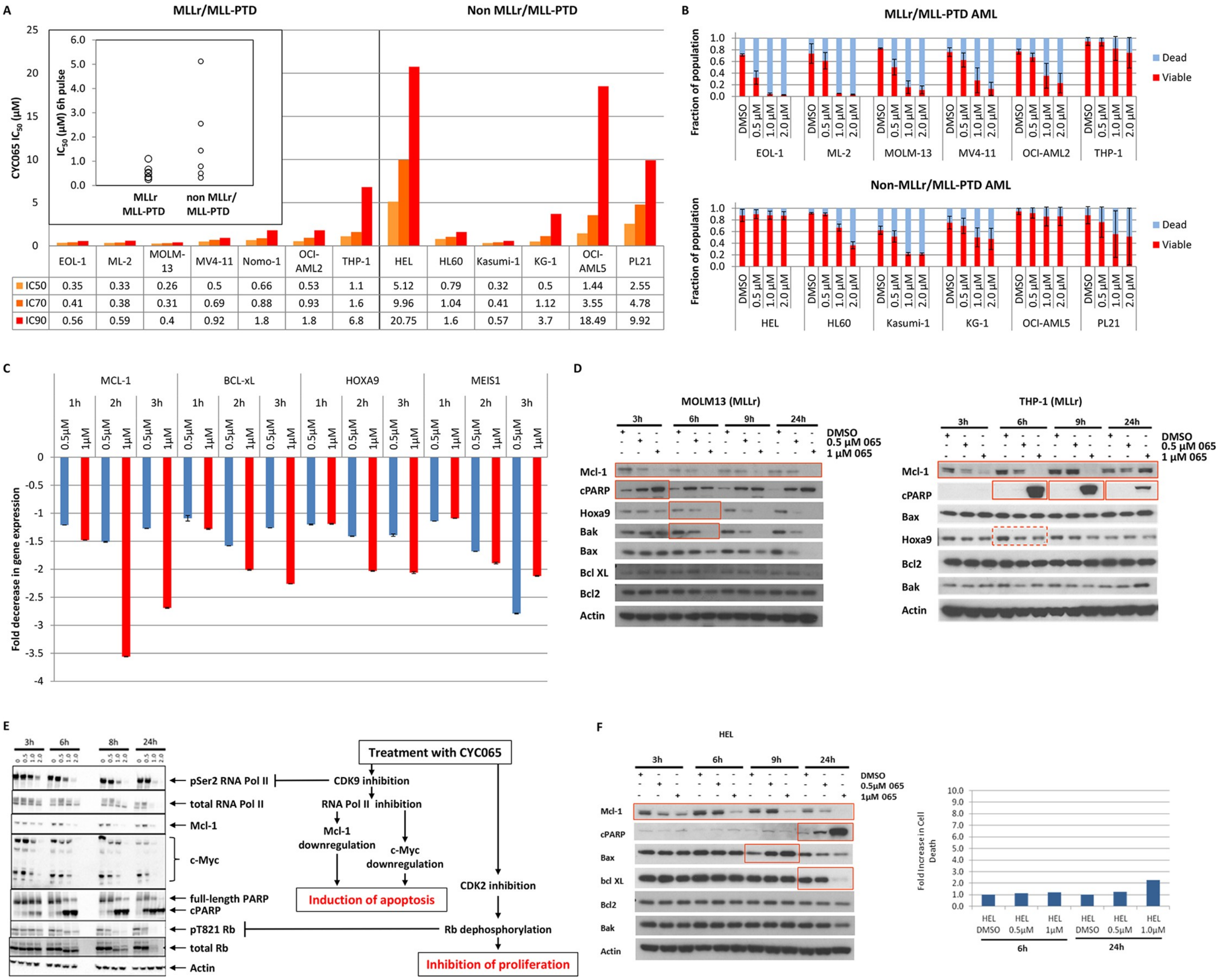

Fig 4. Reliance of AML on MCL1 confers sensitivity to fadraciclib. A. Graph depicting $IC_{50/70//90}$, values determined by resazurin reduction cytotoxicity assay after a 6 h pulse treatment with fadraciclib (CYC065) followed by 66h of release. The graph indicates the average of at least 3 independent experiments. Inset: Comparison of IC_{90} values for a 6 h pulse with fadraciclib (CYC065) in cell lines with or without *MLL*r/*MLL*-PTD (p = 0.03). **B.** Robust induction of cell death in AML cell lines, especially those with *MLL*r/*MLL*-PTD. Viability was assessed at 48 h following a 6 h pulse with fadraciclib (CYC065) at the indicated concentrations using Viacount assay. Error bars denote SD. **C.** Impact of fadraciclib (CYC065) on MLL-mediated transcription. Gene expression was measured by quantitative real-time PCR and normalised to B2M, housekeeping gene. THP-1 cells were treated with 0.5 or 1.0 μM fadraciclib (CYC065), and then harvested at 1, 2 or 3 h to examine the effect of fadraciclib (CYC065) treatment on gene expression. The fold decrease in gene expression of the MLL-mediated genes, *HOXA9* and *MEIS1*, is compared with *MCL1*. Error bars denote SD. **D.** Examining the mechanism of action of fadraciclib (CYC065) in AML cell lines with *MLL*r/*MLL*-PTD. MOLM-13 and THP-1 are AML cell lines were treated with 0.5 μM or 1 μM fadraciclib (CYC065) for up to 24 h. The protein expression of BCL2 family members, HOXA9 and cleaved PARP were determined by Western blotting. **E.** Deciphering the mechanism of action of fadraciclib (CYC065) in Kasumi-1 (non *MLL*r/*MLL*-PTD cell line). Cells were treated with 0.5–2.0 μM fadraciclib (CYC065) for up to 24 h, and the expression levels of key proteins were examined by Western blotting. RNA pol II CTD pSer2 is a direct substrate of CDK9 and RB pThr821 is a proposed CDK2 site. Actin is included as a loading control. Samples for 2 μM fadraciclib (CYC065) at 24h not included in the total RB and RB pThr821 Western blots. **F.** Examining the expression levels of BCL2 family members and induction of apoptosis in the resistant HEL cell line. HEL cells were exposed to 0.5 μM or 1.0 μM fadraciclib (CYC065) for up to 24 h, and the expression levels of several BCL2 family members and the cleavage of PARP (Ab detecting both total and cleaved PARP Santa Cruz (Insight) sc-7150) were examined by Western blotting. Viability of HEL cells was also measured at 6 h and 24 h.

https://doi.org/10.1371/journal.pone.0234103.g004

Of the AML cell lines without *MLL*r/*MLL*-PTD, three were as sensitive to fadraciclib as were the *MLL*r/*MLL*-PTD cell lines. However, three lines (HEL, OCI-AML5 and PL21) were

significantly less sensitive to fadraciclib than all other AML cell lines, with IC_{90} values in the range of 10 to 20 μM (see also section: Potential Patient Stratification Markers).

To determine whether fadraciclib caused cell growth arrest or cell death in individual AML cell lines, a Viacount assay was used to quantify the number of viable and dead cells after 6 h pulse treatment with fadraciclib (Fig 4B). The number of dead cells at different concentrations correlates well with the $IC_{50/70/90}$ values determined in the cytotoxicity assay, suggesting that the predominant consequence of fadraciclib treatment is cell death rather than growth arrest. The Viacount data also highlight that the sensitive cell lines undergo apoptosis, reaching almost 100% non-viable cells with only 1 μM fadraciclib. In the less sensitive cell lines, apoptosis is triggered to a lesser extent, for example reaching approximately 50% in KG-1 and with no appreciable increase in apoptosis in HEL with up to 2 μM fadraciclib.

The impact of fadraciclib on the cell cycle was also examined by propidium iodide staining and flow cytometry (S3 Fig) which confirmed a robust increase in the sub-G1 fraction in sensitive cell lines ($\geq$ 0.5 μM fadraciclib), indicative of induction of cell death. These data are consistent with the expected outcome of CDK9 inhibition and demonstrate that short exposures to fadraciclib are capable of producing commitment to cell death in target cells. These findings also imply that continuous target inhibition may not be required for anti-cancer activity *in vivo*.

Characterization of fadraciclib mechanism of action in AML cell lines with or without *MLL*r/*MLL*-PTD

MLL binds to promoters of *HOX* genes, such as *HOXA*7 and *HOXA*9, to maintain their expression [49]. These HOX proteins regulate hematopoiesis and are normally expressed only in early hematopoietic progenitors. MLL fusion proteins directly and constitutively up-regulate *HOX* expression via recruitment of P-TEFb (CDK9/cyclin T), DOT1 and other proteins involved in transcriptional elongation [48]. Persistent expression of *HOX* genes along with expression of another up-regulated HOX cofactor, MEIS1, appears to be necessary and sufficient to cause and maintain leukemia [49]. CDK9 inhibition may therefore be expected to be highly effective in CDK9/MLL-driven leukemogenesis via inhibition of leukemogenic transcription [50].

To determine the effect of fadraciclib on MLL-driven transcription, the mRNA levels of *HOXA9* and *MEIS1* were measured in THP-1 cells treated with 0.5 or 1 μM fadraciclib for up to 3 h (Fig 4C). Fadraciclib inhibited *HOXA9* and *MEIS1* transcription similarly to *MCL1*.

The impact of fadraciclib on HOXA9 protein levels was then examined in THP-1 and MOLM-13 cells by immunoblotting (Fig 4D). HOXA9 was decreased by fadraciclib treatment, beginning at 6 h. Interestingly, HOXA9 levels failed to recover in MOLM-13, a sensitive *MLL*r cell line, whereas in THP-1 cells loss of HOXA9 was both modest and transient, and was equivalent to levels in control cells at 24 h.

Significantly, MCL1 followed a very similar pattern to HOXA9 in these two cell lines, with its protein levels remaining depleted in MOLM-13 but being restored to levels similar to or even exceeding levels in the DMSO vehicle control-treated THP-1 cells. These findings are also reflected in the pattern of cleaved PARP. In the sensitive MOLM-13 cell line, there was a robust concentration-dependent increase in cleaved PARP at 3 h, which was maintained throughout the duration of the experiment. In contrast, robust increases in cleaved PARP were observed only in THP-1 cells at 1 μM fadraciclib and the levels appeared to be greatly diminished by 24 h, despite the continued presence of the drug. This apparently transient apoptotic response hints at a possible recovery or compensatory mechanism in less sensitive cells, which can overcome–to some extent–the effects of fadraciclib, and could explain the reduced sensitivity of this cell line to CDK9 inhibition.

Asynchronously growing Kasumi-1 cells (non *MLL*r/*MLL*-PTD), which were highly sensitive to fadraciclib, were treated with increasing concentrations of fadraciclib for up to 24 h and analyzed by Western blotting (Fig 4E). As expected, the CDK9 inhibitory activity of the compound resulted in a rapid decrease of RNA pol II CTD pSer2, MCL1 and MYC protein levels, observed after only 3 h of treatment. The latter finding is consistent with a very short half-life for MYC of less than 20 minutes [51]. Downregulation of the pro-survival proteins preceded the accumulation of p85 PARP cleavage product, a marker of apoptosis, which was evident at 3 h and more pronounced 6 h after treatment initiation. A decrease in RB pThr821 level, related to CDK2 inhibitory activity, was detected at 6 h. This coincided with a band shift for both RB pThr821 and total RB protein, indicating dephosphorylation [52]. Depletion and subsequent loss of RB protein was most evident at 8 h and 24 h. Effects of fadraciclib on CDK2 and CDK9 substrates were sustained to the end of the period tested (24 h) as was observed earlier in Colo205 colon cancer cells.

In summary, the core biochemical events occurring in all cancer cell lines examined appear to be similar, involving loss of RNA pol II CTD pSer2, MCL1 and induction of apoptosis. In addition, AML cell lines carrying *MLL*r/*MLL*-PTD appear to show loss of HOXA9, which may also contribute to the loss of viability.

Potential patient stratification markers

Hematological malignancies, such as AML, have been demonstrated to be very sensitive to MCL1 downregulation resulting in a rapid apoptotic response [17]. In our panel, the majority of AML cell lines were very sensitive to fadraciclib, undergoing rapid apoptosis. The presence of the *MLL*r/*MLL*-PTD appears to enrich for sensitivity, although it is not apparent from the analysis of the biochemical events in cell lines with and without *MLL*r/*MLL*-PTD why that is the case. In addition, within the non *MLL*r/*MLL*-PTD group, further markers would be beneficial to differentiate responders from non-responders in this subgroup.

As a first step to understanding the inherent resistance of certain cell lines to fadraciclib, biochemical events were examined in the highly insensitive HEL AML cell line following compound treatment (Fig 4F). Interestingly, similar effects of fadraciclib on MCL1 were observed in this cell line as with the sensitive lines, and at similar concentration levels. However, appearance of cleaved PARP was restricted to the 24 h time-point and was most apparent at 1 μM fadraciclib. Although MCL1 was depleted throughout the duration of the experiment, this alone did not appear to be sufficient to trigger apoptosis in the HEL cell line. However, depletion of BCL2L1 and increase in BAX resulting from a more prolonged treatment with fadraciclib coincided with modest induction of cell death (Figs 4F & S3). This observation is consistent with the reported dependence of HEL cells on BCL2L1 rather than MCL1 for survival [17]. MCL1 belongs to the BCL2 family of proteins which regulate apoptosis, primarily at the mitochondria through the intrinsic apoptotic pathway [53]. These data suggest an important interplay between various components of the BCL2 family in determining cellular responses to MCL1 loss. This indicates that the balance of anti-apoptotic (e.g. MCL1, BCL2, BCL2L1) and pro-apoptotic (e.g. BAX and BAK) proteins might determine sensitivity to transcriptional inhibitors affecting this apoptotic pathway.

To investigate this further, untreated AML cell lysates were analyzed by immunodetection for levels of MCL1, BCL2, BCL2L1, BAX and BAK (Fig 5A). Higher sensitivity of *MLL*r/*MLL*-PTD cell lines appears to correspond with high levels of the pro-apoptotic protein BAX. Interestingly, THP-1, the least sensitive *MLL*r/*MLL*-PTD cell line of this group, had comparatively low levels of BAX pro-apoptotic protein compared with all other *MLL*r/*MLL*-PTD cell lines.

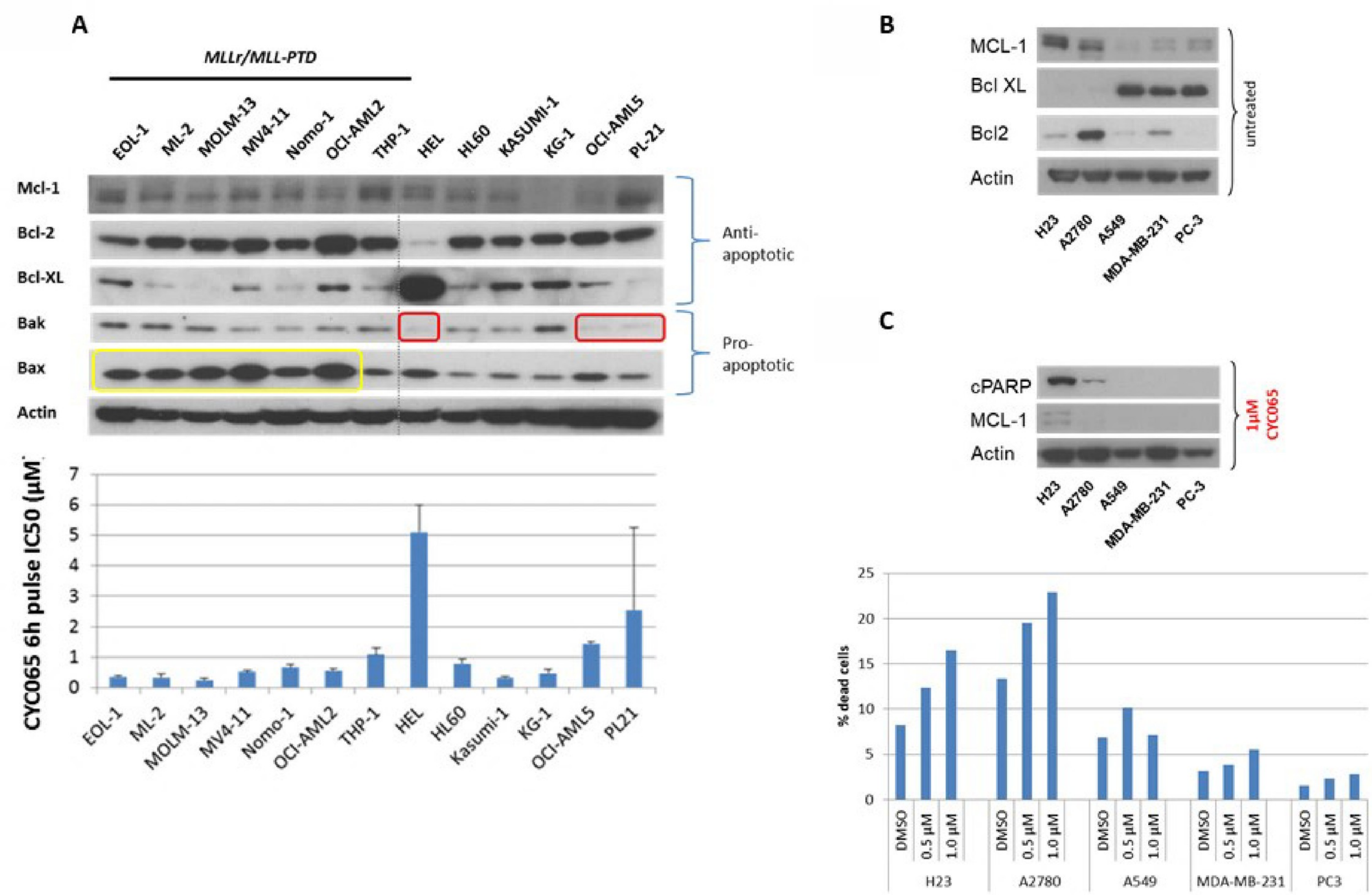

Fig 5. BCL2 family member expression correlates with sensitivity to fadraciclib (CYC065). A. Steady state levels of BCL2 family members in the human AML cell line panel. Lysates were prepared from actively proliferating cell lines and the expression of various BCL2 family members was examined by Western blotting. **B.** Steady state levels of BCL2 family members in selected solid tumour cell lines. **C.** Induction of cell death. Cleavage of PARP was measured by Western blotting (upper) and % dead cells was assessed by Viacount viability assay at 6 h and 24 h.

https://doi.org/10.1371/journal.pone.0234103.g005

In terms of the non *MLL*r/*MLL*-PTD cell lines, HEL, the least sensitive of all AML cell lines tested, had substantially high levels of BCL2L1, which can functionally substitute for MCL1 in binding and neutralizing pro-apoptotic BCL2 family members [44]. High BCL2L1 level was also accompanied by a very low level of the pro-apoptotic protein BAK in this cell line. Relatively low levels of BAK were also observed in two other cell lines that were less sensitive to fadraciclib, OCI-AML5 and PL-21.

It has been reported that apoptosis mediated by MCL1 depletion requires BAK [6]. It thus seems plausible that in the absence of BAK, cells may be more resistant to apoptosis in response to MCL1 depletion [6]. The comparison of IC_{50} values with BAK levels suggests a reciprocal relationship between these two parameters (Fig 5A). These findings suggest that the inherent resistance of HEL and the other two non *MLL*r/*MLL*-PTD cell lines might be related to the low level of the pro-apoptotic protein BAK and are consistent with previous findings [6,44]. For instance, Wei et al. [6] established that, out of over 40,000 genomic features measured, the top feature that predicted sensitivity to transcriptional inhibitor compounds was low expression of *BCL2L1*. They also determined that BAK deficiency protected cells from these compounds.

To expand on the AML findings, human solid tumor cell lines were selected from a larger panel used to characterize dinaciclib-induced apoptosis [44]. The selected cell lines were A2780 (ovarian cancer), PC3 (prostate cancer), MDA-MB-231 (breast cancer) and A549 and H23 NSCLC cell lines. H23 cells have been described in the literature as MCL1-dependent [54]. Fadraciclib sensitivity of these cell lines was determined by comparing IC_{50} values obtained with a 6 h pulse versus continuous treatment for 72 h (S4 Fig). A2780 and H23 cells were highly sensitive to fadraciclib, yielding submicromolar IC_{50} values upon exposure to a short pulse. In contrast, the A549, MDA-MB-231 and PC3 cancer cell lines were sensitive to fadraciclib only after more prolonged treatment.

Untreated cell lysates from these solid tumor cell lines were then examined for expression of MCL1, BCL2L1 and BCL2 to highlight any differences in expression pattern between the two sensitive and the three less sensitive lines (Fig 5B). As expected, the two sensitive cell lines showed high levels of MCL1 and negligible levels of BCL2L1. The less sensitive cell lines displayed the reverse pattern, with substantial levels of BCL2L1 and negligible levels of MCL1. BCL2 was variably expressed and showed no correlation with sensitivity to fadraciclib in this panel. Consistent with observations in the literature for other transcriptional inhibitors [44], it appears in this sample of solid tumors that high expression of MCL1, or indeed a high MCL1: BCL2L1 ratio may confer sensitivity to fadraciclib. Analysis of mRNA levels similarly confirmed that high *MCL1*:*BCL2L1* ratio confers sensitivity to fadraciclib (S5 Fig). Treatment of all five solid tumor cell lines with 1 μM fadraciclib confirmed that only A2780 and H23 cells showed any evidence of apoptosis as determined by appearance of cleaved PARP, accompanied by loss of MCL1, and increase in the proportion of dead cells (Fig 5C).

Fadraciclib combines synergistically with BCL2 inhibitors in AML and ALL cell lines

There are multiple examples of human cell lines that are dependent on MCL1 for survival [see introduction and refs 6,8,9,10,11,12]. These cell lines are highly sensitive to transcriptional inhibition by compounds such as fadraciclib and are characterized by high MCL1:BCL2L1 ratios, and/or detectable levels of BAK. In contrast, there are several cell lines whose survival depend on other BCL2 family members, such as BCL2 or BCL2L1, or in fact may be co-dependent on several anti-apoptotic members. In the latter cell lines, simultaneous inhibition of more than one anti-apoptotic family member may provide an effective mechanism to ablate tumor cells (Fig 6A). To test this hypothesis using cancer cell lines identified in the present study to be less sensitive to fadraciclib, we determined the effects of fadraciclib combined with BCL2 inhibitor venetoclax (ABT-199), or BCL2/BCL2L1/BCL2L2 (bcl-w) inhibitors ABT-263 or ABT-737 in THP-1 (Fig 6B). Similar results were observed with two ALL cell lines, Jurkat and SEM (data not shown).

THP-1 cells were treated in a 7 x 7 matrix of concentrations spanning the IC_{50} value of each compound: fadraciclib (0.43 μM), ABT-199 (0.06 μM), ABT-263 (0.27 μM) and ABT-737 (0.43 μM). Cells were treated concomitantly for 72 h and compared with suitable controls of cells treated with the individual compounds alone. Combination Index (CI) values were calculated using the method of Chou and Talalay [36]. Fadraciclib combined synergistically with venetoclax, ABT-263 and ABT-737 with median CI values of 0.76, 0.74 and 0.57, respectively. Similar results were observed in HEL cells, except that much higher concentrations of venetoclax were required to observe synergy (data not shown). This suggests that high venetoclax concentrations may be necessary to inhibit BCL2L1 as well as BCL2 in this cell line consistent with the observation that HEL cells show significant overexpression of and is dependent on BCL2L1.

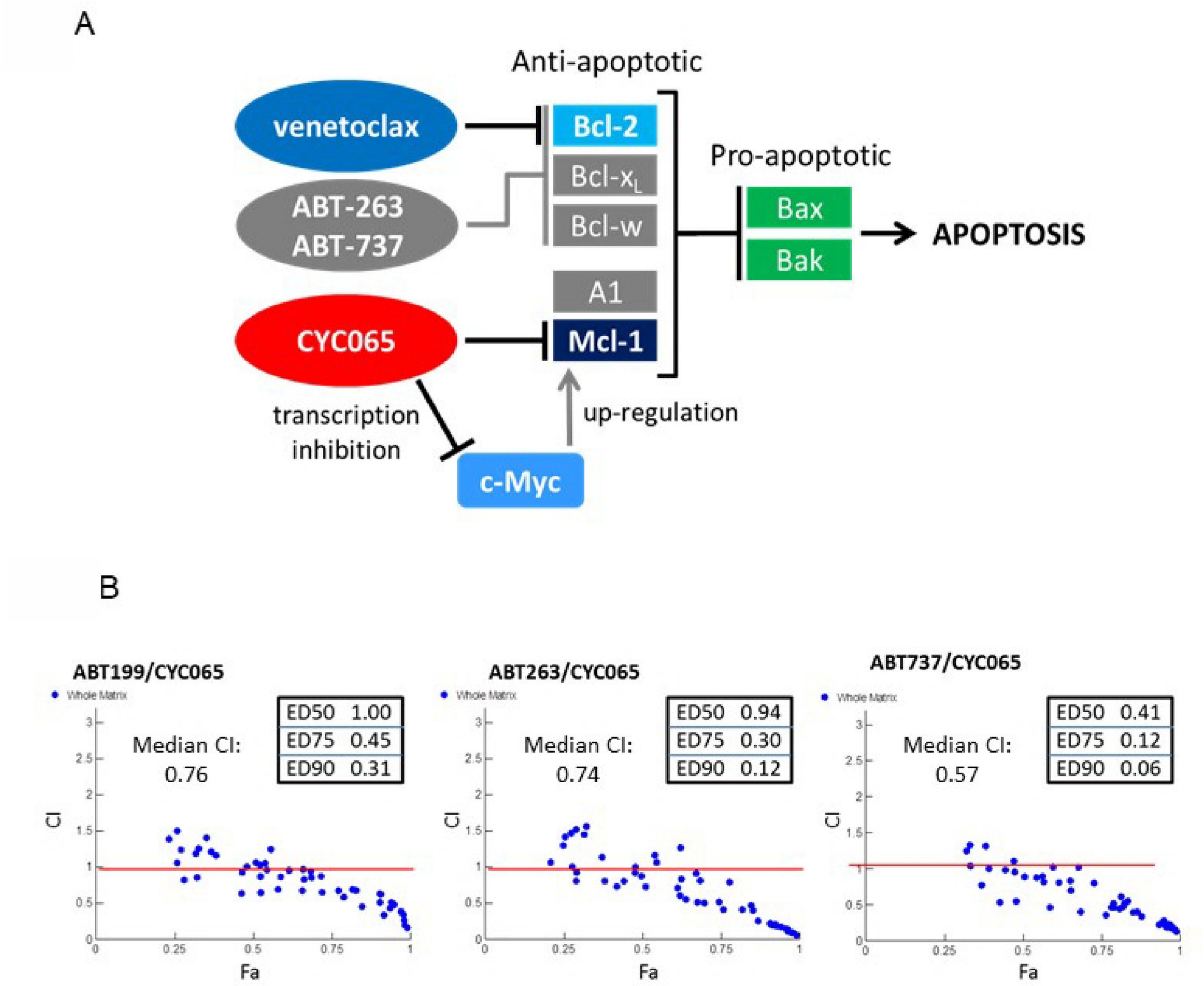

Fig 6. Dual targeting of MCL1 and BCL2 is highly synergistic at inducing cell death. A. Schematic diagram of BCL2 family members, interaction and inhibitors. Venetoclax (ABT-199) is a selective BCL2 inhibitor, whereas ABT-263 and ABT-737 inhibit BCL2, BCL2L1 and BCL2L2. Bcl-x_L = BCL2L1, Bcl-w = BCL2L2, A1 = BCL2A1 / Bfl-1. **B.** Fadraciclib (CYC065) combines synergistically with inhibitors of the BCL2 family in THP-1 cells. y axis–combination index (CI); x-axis–fraction affected (Fa). CI values were calculated using the method of Chou and Talalay [36]. CI values of 0.9–1.1 indicate an additive drug interaction, CI values of >1.1 are considered antagonistic and CI values <0.9 are considered synergistic.

https://doi.org/10.1371/journal.pone.0234103.g006

Fadraciclib has potent anti-AML activity *in vivo*

Overall, the cellular pharmacology data showed that fadraciclib effectively downregulates pro-survival and leukemogenic proteins and that short pulse treatment is sufficient to substantially decrease cell viability of the majority of AML cell lines tested via apoptotic induction. To test if these effects will translate into anti-leukemic activity *in vivo* we used two subcutaneous xeno-graft models EOL-1 (with *MLL*-PTD) and HL60 (non *MLL*r/*MLL*-PTD AML with *MYC* amplification).

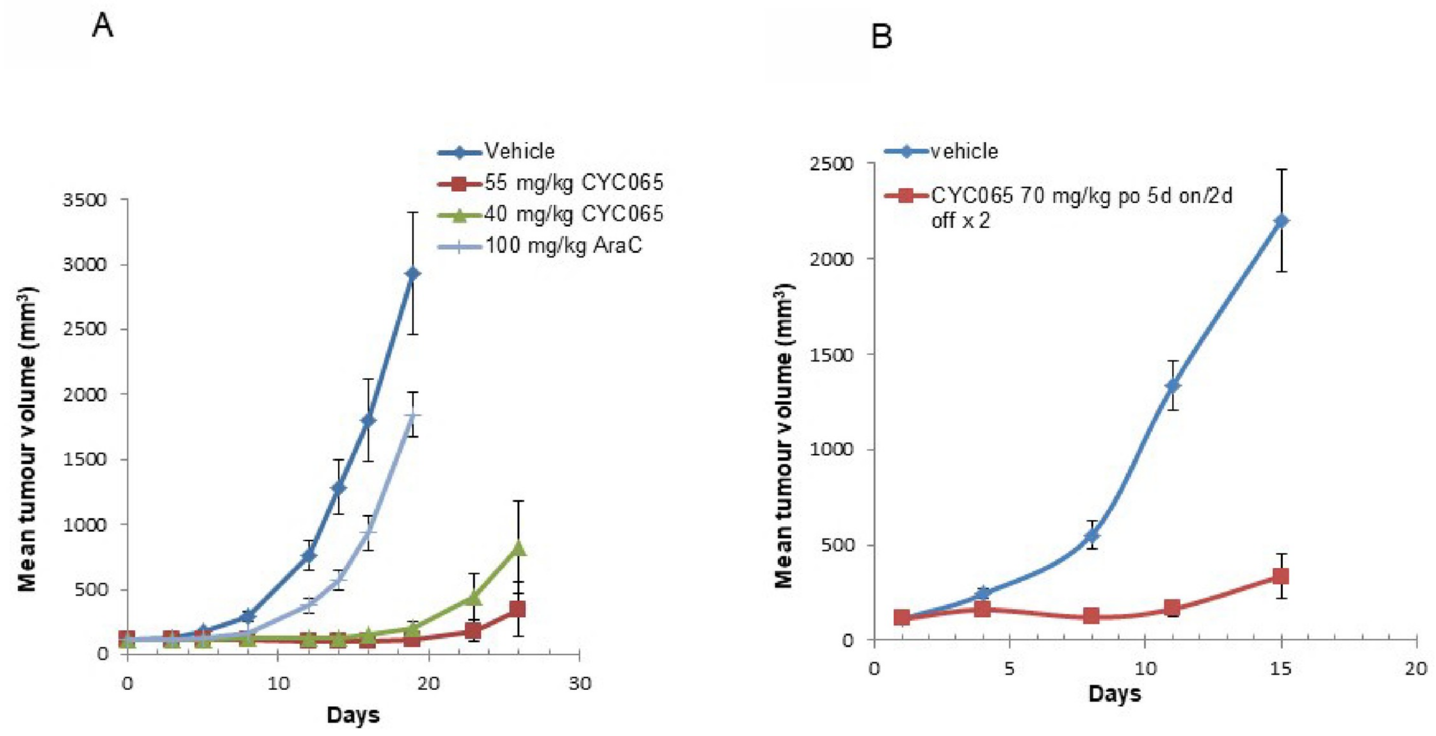

Fig 7. *In vivo* efficacy of fadraciclib (CYC065) in AML xenograft models. A. Potent anti-tumour activity of fadraciclib (CYC065) in the EOL-1 *MLL*-PTD AML xenograft model. Fadraciclib (CYC065) was administered orally at 40 or 55 mg/kg qd x 5 for 2 weeks. AraC was administered using an optimised schedule for this model, consisting of 100mg/kg i.p. on days 1–5. The median TGI achieved on day 9 was 97% and 95% for 55 and 40 mg/kg fadraciclib (CYC065), respectively, compared with 41% for AraC. **B.** Anti-tumour activity in HL60 (*MYC* amplified non-*MLL*r/*MLL*-PTD) AML xenograft model. Fadraciclib (CYC065) was administered at 70 mg/kg orally over 5 days for 2 weeks, and delayed the growth of HL60 xenografts (T-C >12.6 days) and TGI on day 11 was 90%, which was significantly different to the vehicle control group (p<0.001). Compound treatment was well tolerated in both models at all doses with a mean body weight loss of 8% at the highest dose.

https://doi.org/10.1371/journal.pone.0234103.g007

SCID mice with established subcutaneous EOL-1 tumors were randomized into groups of 6 animals and assigned to vehicle control, fadraciclib at 40 or 55 mg/kg, or cytarabine 100 mg/kg (Fig 7A). Cytarabine (a nucleoside analogue, incorporated into DNA) was used as a positive control as this is one of the main chemotherapeutic agents used in pediatric and adult patients with AML and ALL, including those with MLL aberrations. Fadraciclib administration at 40 and 55mg/kg resulted in 97% and 100% tumor growth inhibition (TGI), respectively, on Day 19 (study completion; p<0.0001 versus vehicle control). In comparison, cytarabine treatment inhibited tumor growth by 38%. Overall, the treatments were well tolerated with only transient body weight loss of 2% and 3% in the fadraciclib 55 mg/kg and cytarabine groups respectively (data not shown).

HL60 was selected as a representative, sensitive, non *MLL*r/*MLL*-PTD AML cell line. Nude mice with subcutaneous HL60 tumors were randomized into groups of 10 animals and assigned to vehicle control or fadraciclib treatment groups (Fig 7B). Fadraciclib administration at 70mg/kg delayed the growth of HL60 leukemia xenografts with a T-C value of more than 12.6 days. Two animals achieved tumor regression and one was determined to be tumor-free. TGI on Day 11 was 90%, significantly different versus vehicle control (p< 0.001). An average of 8% body weight loss was observed in the treatment group (data not shown).

In summary, fadraciclib was well tolerated and showed promising efficacy in two subcutaneous AML xenografts as a single agent in agreement with the essential role for MCL1 in AML survival.

Discussion

We report the first disclosure for the chemical structure, cellular mechanistic properties and promising therapeutic activity of the CDK2 and CDK9 clinical candidate fadraciclib. The results shown are consistent with the principal anti-cancer effects of the drug being mediated by inhibition of CDK2 and CDK9, for which IC50 values are 4.5 and 26 nM respectively. In addition to CDK2 and CDK9, fadraciclib also inhibits CDK3 and CDK5 with IC50 values of 28.9 and 20.5 nM. However, the data from genome-wide CRISPR-Cas9 screens indicate that in almost all cancer cell lines tested, including those used in the present study, genetic loss of CDK3 and CDK5 has a minimal impact on cell survival, in clear contrast to the marked dependency of cancer lines following genetic knockdown of CDK2 and especially CDK9.

The modest clinical efficacy of the early transcriptional CDK inhibitors, alvocidib, seliciclib and others, was likely due to their suboptimal pharmacokinetic profile, broad CDK inhibitory activity or lack of potency against their primary target(s). This prompted the design of more potent and selective compounds with improved metabolic stability [32,33]. We show that fadraciclib demonstrates improved kinase inhibitory potency *in vitro* against its primary CDK targets–CDK2 and CDK9 (Fig 1B)–as well as anti-cancer activity in *in vitro* and *in vivo* models (Fig 7). Fadraciclib has now completed the first part of a first-in-human study in advanced solid tumors (NCT02552953). In patient PBMCs, fadraciclib treatment at the recommended phase 2 dose (RP2D) caused durable suppression of MCL1 beyond 24 h, suggesting prolonged target engagement, and was generally well tolerated [55]. The durable reduction of MCL1 expression in the majority of patients treated at the RP2D appears to be an important differentiator for fadraciclib. Data on previous CDK inhibitors including alvocidib, dinaciclib or seliciclib indicated that decreases in MCL1 protein levels in patient peripheral blood samples were only transient [21,22]. In addition to drug exposure kinetics, the kinase inhibitory profile may also contribute to the lack of durable target inhibition seen with these agents. A BRD4-mediated compensatory mechanism has been described in response to CDK9 inhibition [56] which leads to upregulation of MYC and MCL1 at suboptimal drug concentrations. Since BRD4 kinase activity is inhibited by CDK7 mediated phosphorylation, CDK9 inhibitors cross-reacting with CDK7 are likely to be more limited by this compensatory effect. Fadraciclib has the advantage of being selective for CDK9 versus CDK7. In addition, the potent CDK2 inhibitory activity may provide further advantage. Choudhary et al. [27] demonstrated that cyclin E/Cdk2-dependent phosphorylation of MCL1 residues on its PEST domain resulted in increased MCL1 stability (Thr92, and Thr163) and BIM binding (Ser64). This suggests that the effect of fadraciclib on MCL1 may operate at several levels: inhibition of transcription, decrease of protein stability and reduced protein function. Although, the latter two need to be investigated, Poon et al. [43] demonstrated in a comparative study with selective CDK7, CDK9, CDK4/6 and pan CDK inhibitors that fadraciclib, a CDK2/9 inhibitor, was the most effective agent at downregulating MCL1 and MYC at growth inhibitory concentrations in neuroblastoma models.

MCL1 amplification and overexpression have been reported in various human cancers, including hematological malignancies and solid tumors (e.g., NSCLC, breast, ovarian, prostate and pancreatic cancers) [57–60]. MCL1 has been shown to be both an intrinsic and acquired resistance factor, limiting the efficacy of various standard anticancer drugs. This made the anti-apoptotic protein an attractive cancer therapeutic target and several classes of agents, including CDK9 and direct MCL1 inhibitors, are now being tested in clinical trials [reviewed in refs: 61,62].

AML is a heterogeneous hematologic malignancy with unmet medical need. Targeting the CDK9 pathway, which is dysregulated in AML, is an attractive approach leading to

downregulation of genes regulated by super enhancers such as *MCL1*, *MYC* and cyclin D1 [reviewed in 3]. Indeed, we show here that fadraciclib is very effective at inhibiting CDK9 activity and inducing apoptosis in most AML cell models. *MLL*r AML cell lines were particularly sensitive to fadraciclib. *MLL*r are associated with a highly aggressive subtype of acute leukemia with a very dismal prognosis. The coupling of the 5' prime region of *MLL* to a variety of partner genes generates a fusion protein that recruits the DOT1L methyltransferase and RNA pol II together with the positive transcription elongation factor b (P-TEFb) (CDK9/cyclin T). This stimulates transcriptional elongation at *MLL* loci even in the presence of differentiating stimuli, leading to leukemogenesis [48] and suggests that transcriptional inhibition will be effective in *MLL*r genetic background. This has been adequately demonstrated in preclinical studies using knockdown of DOT1L, conditional knockout mice or the clinical small molecule DOT1L inhibitor, EPZ-5676, as well as several of the small molecule CDK9 inhibitors, such as alvocidib and dinaciclib [5,50,63]. The majority of *MLL* fusion-transformed cells were more sensitive to CDK9 inhibitors than the non *MLL*r cell lines. Similarly, in comparison with primary hematopoietic cells, those transduced with *MLL-ENL* were more sensitive to alvocidib and alsterpaullone [50].

Sensitivity to fadraciclib was increased in AML and ALL cell lines carrying *MLL*r/*MLL*-PTD (Fig 4A and 4B, S2 Fig and S7 Table), and this may be due to dependence on the MLL-mediated transcription of *HOXA9*/*MEIS1*. Although a reduction in the mRNA levels of both *HOXA9* and *MEIS1* was observed (Fig 4C), the loss of HOXA9 protein occurred concurrently with (rather than preceding) the appearance of cleaved PARP (Fig 4D). This in turn suggests that loss of HOXA9 is not the primary driver of apoptosis in these cells. Interestingly, we observed higher expression levels of pro-apoptotic BAX in all sensitive *MLL*r/*MLL*-PTD cell lines (Fig 5A). When coupled with the fadraciclib-mediated loss of MCL1 and release of BAK, the second pro-apoptotic member, could account for *MLL*r/*MLL*-PTD cells becoming highly apoptotic (Fig 4B).

Reduced sensitivity to fadraciclib was associated with overexpression of BCL2L1 (Fig 5A and 5C) and loss of BAK (Fig 5A and 5B). These findings are to be expected in the mitochondrial apoptosis pathway as MCL1 and BCL2L1 are functionally redundant in their ability to bind and sequester pro-apoptotic BAK, and in the absence of BAK, apoptosis is compromised. These results are consistent with larger studies using other methods to deplete MCL1 [6,44].

In a large chemical genomics study of more than 2000 compounds screened in over 100 human breast and lung cancer cell lines, low BCL2L1 expression was identified as the best predictor of sensitivity to transcriptional repressors [6]. Thus, ectopic expression of BCL2L1 rescues cancer cells from transcriptional repressor treatment and tumors with high levels of BCL2L1 or loss of BAK are resistant to MCL1 depletion. The contribution of elevated BCL2L1 expression to driving resistance to MCL loss was further confirmed in a large genetic knockdown screen of a cell panel comprising 100s of cell lines from different tumor types [64]. Sensitivity to dinaciclib in a large panel of solid tumor cell lines was associated with a high MCL1: BCL2L1 ratio, suggesting that patients could be pre-selected on the basis of this parameter or perhaps the presence of *MCL1* amplification (associated with a high MCL1:BCL2L1 ratio) [44].

For those tumors not exquisitely dependent on or sensitive to the depletion of MCL1, e.g. those with some reliance upon other BCL2 family members, including some AML cell lines reported here (HEL, THP-1, PL21), a combination strategy has worked very well in preclinical experiments. Dual targeting of the two main pro-survival pathways using ABT-263/ABT-737 or venetoclax, in combination with depletion of MCL1 is extremely efficient at inducing widespread apoptosis (Fig 6A and 6B). The benefit of dual targeting of both BCL2 family members

is therefore promising in AML and other hematological malignancies and is now being investigated in the clinic.

CDK2 inhibition may also contribute to the induction of apoptosis as phosphorylated RB is protective against apoptosis. This implicates RB dephosphorylation directly in triggering of cell death. Loss of RB phosphorylation could be a consequence of CDK2 inhibition and/or activation of an RB-directed phosphatase (through loss of PNUTS), which has been shown to be required for apoptosis under certain conditions [65,66]. These two events, loss of RB phosphorylation on purported CDK2 sites and rapid loss of the *PNUTS* transcript via CDK9 inhibition, have both been detected with seliciclib and fadraciclib at relevant time-points and concentrations. The loss of *PNUTS* transcript follows similar kinetics to MCL transcript loss whereas the loss of RB phosphorylation is delayed to approximately 6 h or later (Fig 4E).

The CDK2 inhibitory activity of fadraciclib is of particular relevance for tumors with amplified/overexpressed cyclin E, which are characterized by poor prognosis and drug resistance. Schraml et al. [67] analysed cyclin E alterations in tissue microarrays consisting of 3670 primary tumors from 128 different tumor types, 709 metastases, and 354 normal tissues. Cyclin E amplifications were reported in 15 tumor types such as bladder, colon, esophageal, gastric, ovarian cancers. Overexpression of cyclin E was observed in 48 tumor types such as breast, cervix, endometrium, ovary, esophagus, stomach, colon, gall bladder, liver, prostate, testis, urinary bladder, head and neck, lung, bone, skin, thyroid, lymphatic system and others.

There is evidence that Her2+ breast cancer (BC) cell lines resistant to trastuzumab show amplification and overexpression of cyclin E. These lines are more sensitive to inhibition of CDK2/9 by fadraciclib, which caused both cell cycle arrest and apoptosis [25]. Similar results have been found in uterine serous carcinoma (USC) primary samples. Almost half of all USC samples examined showed amplification of cyclin E, and these were considerably more sensitive to inhibition of CDK2/9 by fadraciclib than their non-amplified counterparts [26]. In both, Her2+ BC and USC, models fadraciclib and trastuzumab had synergistic effect *in vitro* and *in vivo* [25,26]. Interestingly, lower concentrations of fadraciclib, caused predominantly G1 arrest, consistent with the enhanced potency of fadraciclib against CDK2 over CDK9. Thus in the absence of a strong apoptotic phenotype, the impact of fadraciclib on CDK2 and the cell cycle may become more evident and relevant.

Cyclin E overexpression confers rsistance also to CDK4/6 inhibitors [68]. A recent analysis of samples from patients on the PALOMA-3 trial indicated that high levels of cyclin E1 were associated with relative resistance to the CDK4/6 inhibitor palbociclib [69]. This study provides clinical validation for CDK2/cyclin E as the key bypass kinase of CDK4/6 inhibition and highlights the potential to target CDK2/cyclin E with agents like fadraciclib to overcome resistance. Although CDK2 knockdown can overcome resistance in preclinical models [70], it remains to be confirmed whether inhibition of CDK2/cyclin E kinase activity is also efficacious. Similarly, it will be interesting to establish whether CDK2 inhibitors can substitute for CDK4/6 inhibitors in combination with endocrine therapy [71], or whether the triple combination of CDK4/6 andCDK2 inhibitors plus endocrine therapy may result in more durable clinical responses.

There are many outstanding questions regarding the optimal CDK selectivity profile for oncology therapeutics. As new agents are developed and evaluated in the clinic, it will be essential to determine and collate all relevant parameters. These include potency against all available CDK enzymes *in vitro* and not just a subset, broader kinome profiling and mechanistic cellular properties, together with pharmacokinetic and pharmacodynamic behavior, in order to make valid comparisons and draw conclusions on optimal features, particularly concerning kinase selectivity. Genome-wide profiling of patients and functional profiling of mitochondrial apoptotic priming potential, should also be included, wherever possible, to mine for sensitive

patient populations for each drug or class of drugs. After many years of incomplete understanding of their potential clinical utility, we are entering an exciting period during which the optimal use of transcriptional CDK inhibitors, including fadraciclib, is emerging for the benefit of patients with unmet medical need.

Supporting information

S1 Table. Cell line information. The cell lines utilised in this study are listed alongside various parameters of interest as indicated. Key: y = years, M = male, F = female, PB = peripheral blood, BM = bone marrow, h = hours, TNBC = triple negative breast cancer.
EMT = epithelial-mesenchymal transition. Molecular genetic data listed for the AML cell lines was derived from COSMIC database (http://cancer.sanger.ac.uk/cancergenome/projects/cosmic/) or on the DSMZ website.
(DOCX)

S2 Table. Antibodies used for Western blotting. Antibodies are listed together with the commercial source, catalogue number, dilution used in the study and the species.
(DOCX)

S3 Table. Comparison of CDK profile of seliciclib, CCT068127 and fadraciclib (CYC065). IC_{50} ± SD (μM) for seliciclib, CCT068127 and fadraciclib (CYC065) for CDK2, CDK4, CDK7 and CDK9 showing values presented in Fig 1B.
(DOCX)

S4 Table. IC_{50} values in human colon cancer cell line for seliciclib, CCT068127, fadraciclib (CYC065) and alvocidib (flavopiridol). Colo205 72 h continuous treatment IC_{50} ± SD (μM) for seliciclib, CCT068127, fadraciclib (CYC065) and alvocidib (flavopiridol). Values are the mean of 3 independent experiments, each run in triplicate. Values determined were used to select treatment conditions for western blotting and flow cytometry analysis shown in Fig 1.
(DOCX)

S5 Table. Comparison of the cytotoxicity of seliciclib and fadraciclib (CYC065) in a panel of cell lines. The cell lines included in this study are listed along with the IC_{50} (μM) for seliciclib and fadraciclib (CYC065) after a continuous 72 h treatment. The fold difference in potency between seliciclib and fadraciclib (CYC065) is indicated on the right column.
(DOCX)

S6 Table. Carna Biosciences Kinase Profile Screening Results. Fadraciclib (CYC065) (1μM) was evaluated in a 256-kinase panel at approximately Km[ATP] and showed excellent selectivity. The percent inhibition of each kinase by fadraciclib (CYC065) is indicated in the table. Nine kinases were inhibited by >50% and the IC_{50} values were established against these CDK and CDK-like kinases in a separate assay.
(DOCX)

S7 Table. Comparison of IC_{50} values from a 6 h pulse and continuous 72 h treatments in the AML cell line panel. AML cell lines were incubated with fadraciclib (CYC065) for the indicated duration and IC_{50} values were determined, and compared. Nine out of thirteen cell lines were highly sensitive to fadraciclib (CYC065) and displayed 6 h pulse IC_{50} values similar to their 72 h continuous IC_{50} values. Values are the mean of 3 independent experiments.
(DOCX)

S8 Table. Combination analysis of fadraciclib (CYC065) with BCL2 inhibitors in THP-1 cells. Concomitant treatment with fadraciclib and BCL2 inhibitor venetoclax (ABT199) or

BCL2/BCL2L1 inhibitors ABT263 and ABT737 was performed and analysed as described in Materials and Methods. Average combination index (CI) and SD values are listed.
(DOCX)

S1 Fig. Exploring the kinetics of cellular response to fadraciclib (CYC065) in Kasumi-1 cells. Kasumi-1 cells were treated with 0.5 or 1.0 μM fadraciclib (CYC065) for up to 6 h, with cells harvested every hour for examination of the levels of MCL1 and cleaved PARP by Western blotting (A). Kasumi-1 cells were pulse treated with 0.5 or 1.0 μM fadraciclib (CYC065) for up to 6 h with medium replaced at the indicated times, and then samples harvested at 24 h from the start of treatment to assess viability by Viacount assay (B).
(DOCX)

S2 Fig. *MLL*-ALL is more sensitive to fadraciclib (CYC065) than non-*MLL*r/*MLL*-PTD. An ALL cell line panel was treated with fadraciclib (CYC065) for 10 h then incubated in compound-free medium until viability was assessed at 72 h. All cell lines with *MLL*r/*MLL*-PTD appeared to be more sensitive to fadraciclib (CYC065) than non-*MLL*r/*MLL*-PTD cell lines.
(DOCX)

S3 Fig. Increase in sub G1 in sensitive AML cell lines. AML cell lines were treated with fadraciclib (CYC065) for 16 h at concentrations from 0.1–1.0 μM, then fixed and stained for cell cycle analysis by propidium iodide. The two sensitive cell lines, MOLM-13 and Kasumi-1, both show robust induction in subG1, indicative of cell death, whereas HEL cell line shows only a modest increase in sub G1 after 16 h continuous treatment with the compound.
(DOCX)

S4 Fig. Comparison of IC_{50} values in selected solid tumour cells lines after a 6 h pulse or 72 h continuous treatment with fadraciclib (CYC065). Sensitive cell lines, A2780 and H23, show similar IC_{50} values with short pulse or continuous treatment, whereas, the resistant cell lines A549, MDA-MB-231 and PC3, are only sensitive to prolonged treatment with fadraciclib (CYC065).
(DOCX)

S5 Fig. Sensitive cell lines show a high *MCL1*:*BCL2L1* mRNA ratio. The levels of *MCL1* and *BCL2L1* mRNA as determined by qPCR were examined in selected sensitive and resistant solid tumour cell lines. Sensitive cell lines, H23 and A2780, had high levels of *MCL1* and lower levels of *BCL2L1* –confirming the results obtained by Western blotting.
(DOCX)

S6 Fig. Plot of CERES gene effect scores for knockdown of CDK2, 3, 5 and 9 in cancer cell lines, as a measure of dependency on these genes. Data were obtained from genome-wide CRISPR-Cas9 screens with the Avana sgRNA library in cancer cell lines and deposited as part of the Cancer Dependency Map project (https://depmap.org/portal/; Computational correction of copy number effect improves specificity of CRISPR–Cas9 essentiality screens in cancer cells [72]. A lower CERES score indicates a higher likelihood that the gene of interest is essential in a given cell line. The blue box-whisker plots correspond to data for all of the >700 cancer cells for which data are available in DepMap; the red box-whisker plots correspond to data from cancer cell lines described in S5 Table. The box shows the median value and the interquartile range between the first and third quartiles. The bars show the minimum and maximum range of the population.
(DOCX)

Author Contributions

Conceptualization: Sheelagh Frame, Paul Workman, David Blake, Daniella Zheleva.

Data curation: Sheelagh Frame, Chiara Saladino, Craig MacKay, Paul A. Clarke, David Blake, Daniella Zheleva.

Formal analysis: Sheelagh Frame, Chiara Saladino, Craig MacKay, Edward McDonald, Paul A. Clarke, David Blake, Daniella Zheleva.

Investigation: Sheelagh Frame, Chiara Saladino, Craig MacKay, Butrus Atrash, Peter Sheldrake.

Project administration: David Blake, Daniella Zheleva.

Supervision: Sheelagh Frame, Edward McDonald, David Blake, Daniella Zheleva.

Writing – original draft: Sheelagh Frame, Daniella Zheleva.

Writing – review & editing: Paul A. Clarke, Paul Workman, David Blake.

References

1. Whittaker SR, Mallinger A, Workman P, Clarke PA. Inhibitors of cyclin-dependent kinases as cancer therapeutics. Pharmacol Ther. 2017; 173: 83–105. Review. https://doi.org/10.1016/j.pharmthera.2017.02.008 PMID: 28174091
2. Koumenis C, Giaccia A. Transformed cells require continuous activity of RNA polymerase II to resist oncogene-induced apoptosis. Mol Cell Biol. 1997; 17: 7306–7316. https://doi.org/10.1128/mcb.17.12.7306 PMID: 9372962
3. Boffo S, Damato A, Alfano L, Giordano A. CDK9 inhibitors in acute myeloid leukemia. Journal of Experimental & Clinical Cancer Research. 2018; 37:36 https://doi.org/10.1186/s13046-018-0704-8
4. Doroshow DB, Eder JP, LoRusso PM. BET inhibitors: a novel epigenetic approach. Annals of Oncology. 2017; 28: 1776–1787. https://doi.org/10.1093/annonc/mdx157 PMID: 28838216
5. Daigle SR, Olhava EJ, Therkelsen CA, Basavapathruni A, Jin L, Boriack-Sjodin PA, et al. Potent inhibition of DOT1L as treatment of MLL-fusion leukemia. Blood. 2013; 122: 1017–1025. https://doi.org/10.1182/blood-2013-04-497644 PMID: 23801631
6. Wei G, Margolin AA, Haery L, Brown E, Cucolo L, Julian B, et al. Chemical genomics identifies small-molecule MCL1 repressors and BCL-xL as a predictor of MCL1 dependency. Cancer Cell. 2012; 2: 547–562. https://doi.org/10.1016/j.ccr.2012.02.028 PMID: 22516262
7. Ma H, Seebacher NA, Hornicek FJ, Duan Z. Cyclin-dependent kinase 9 (CDK9) is a novel prognostic marker and therapeutic target in osteosarcoma. EBioMedicine. 2019; 39: 182–193. https://doi.org/10.1016/j.ebiom.2018.12.022 PMID: 30579871
8. Beroukhim R, Mermel CH, Porter D, Wei G, Raychaudhuri S, Donovan J, et al. The landscape of somatic copy-number alteration across human cancers. Nature. 2010; 463:899–905. https://doi.org/10.1038/nature08822 PMID: 20164920
9. Pepper C, Lin TT, Pratt G, Hewamana S, Brennan P, Hiller L, et al. Mcl-1 expression has *in vitro* and *in vivo* significance in chronic lymphocytic leukemia and is associated with other poor prognostic markers. Blood. 2008; 112: 3807–3817. https://doi.org/10.1182/blood-2008-05-157131 PMID: 18599795
10. Wuillème-Toumi S, Robillard N, Gomez P, Moreau P, Le Gouill S, Avet-Loiseau H, et al. Mcl-1 is overexpressed in multiple myeloma and associated with relapse and shorter survival. Leukaemia. 2005; 19:1248–1252.
11. Kaufmann SH, Karp JE, Svingen PA, Krajewski S, Burke PJ, Gore SD, et al. Elevated expression of the apoptotic regulator Mcl-1 at the time of leukemic relapse. Blood. 1998; 91: 991–1000. PMID: 9446661
12. Goodwin CM, Rossanese OW, Olejniczak ET, Fesik SW. Myeloid cell leukemia-1 is an important apoptotic survival factor in triple-negative breast cancer. Cell Death Differ. 2015; 22: 2098–2106. https://doi.org/10.1038/cdd.2015.73 PMID: 26045046
13. Song L, Coppola D, Livingston S, Cress D, Haura EB. Mcl-1 regulates survival and sensitivity to diverse apoptotic stimuli in human non-small cell lung cancer cells. Cancer Biol Ther. 2005; 4:267–276. https://doi.org/10.4161/cbt.4.3.1496 PMID: 15753661

14. Modugno M, Banfi P, Gasparri F, Borzilleri R, Carter P, Cornelius L, et al. Mcl-1 antagonism is a potential therapeutic strategy in a subset of solid cancers. Exp Cell Res. 2015; 332: 267–277. https://doi.org/10.1016/j.yexcr.2014.11.022 PMID: 25486070

15. Besbes S, Pocard M, Mirshahi M, Billard C. The first MCL-1-selective BH3 mimetics have therapeutic potential for chronic lymphocytic leukemia. Crit Rev Oncol Hematol. 2016; 100: 32–36. https://doi.org/10.1016/j.critrevonc.2016.02.003 PMID: 26899021

16. Derenne S, Monia B, Dean NM, Taylor JK, Rapp MJ, Harousseau JL, et al. Antisense strategy shows that Mcl-1 rather than Bcl-2 or Bcl-x(L) is an essential survival protein of human myeloma cells. Blood. 2002; 100: 194–199. https://doi.org/10.1182/blood.v100.1.194 PMID: 12070027

17. Glaser SP, Lee EF, Trounson E, Bouillet P, Wei A, Fairlie WD, et al. Anti-apoptotic Mcl-1 is essential for the development and sustained growth of acute myeloid leukemia. Genes Dev. 2012; 26:120–125. https://doi.org/10.1101/gad.182980.111 PMID: 22279045

18. Kelly GL, Grabow S, Glaser SP, Fitzsimmons L, Aubrey BJ, Okamoto T. Targeting of MCL-1 kills MYC-driven mouse and human lymphomas even when they bear mutations in p53. Genes Dev. 2014; 28: 58–70. https://doi.org/10.1101/gad.232009.113 PMID: 24395247

19. Yoshimoto G, Miyamoto T, Jabbarzadeh-Tabrizi S, Iino T, Rocnik JL, Kikushige Y, et al. FLT3-ITD up-regulates MCL-1 to promote survival of stem cells in acute myeloid leukemia via FLT3-ITD-specific STAT5 activation. Blood. 2009; 114: 5034–5043. https://doi.org/10.1182/blood-2008-12-196055 PMID: 19808698

20. Aichberger KJ, Mayerhofer M, Krauth MT, Skvara H, Florian S, Sonneck K, et al. Identification of mcl-1 as a BCR/ABL-dependent target in chronic myeloid leukemia (CML): evidence for cooperative antileukemic effects of imatinib and mcl-1 antisense oligonucleotides. Blood. 2005; 105: 3303–3311. https://doi.org/10.1182/blood-2004-02-0749 PMID: 15626746

21. Gojo I, Sadowska M, Walker A, Feldman EJ, Iyer SP, Baer MR, et al. Clinical and laboratory studies of the novel cyclin-dependent kinase inhibitor dinaciclib (SCH 727965) in acute leukemias. Cancer Chemother Pharmacol. 2013; 72: 897–908. https://doi.org/10.1007/s00280-013-2249-z PMID: 23949430

22. Bible KC, Lensing JL, Nelson SA, Lee YK, Reid JM, Ames MM, et al. Phase 1 trial of flavopiridol combined with cisplatin or carboplatin in patients with advanced malignancies with the assessment of pharmacokinetic and pharmacodynamic end points. Clin Cancer Res. 2005; 11: 5935–5941. https://doi.org/10.1158/1078-0432.CCR-04-2566 PMID: 16115936

23. Lim S, Kaldis P. Cdks, cyclins and CKIs: roles beyond cell cycle regulation. Development. 2013; 140: 3079–3093. https://doi.org/10.1242/dev.091744 PMID: 23861057

24. Bacevic K, Lossaint G, Achour TN, Georget V, Fisher D, Dulic V. Cdk2 strengthens the intra-S checkpoint and counteracts cell cycle exit induced by DNA damage. Scientific Reports. 2017; 7: 13429–13443. https://doi.org/10.1038/s41598-017-12868-5 PMID: 29044141

25. Scaltriti M, Eichhorn PJ, Cortés J, Prudkin L, Aura C, Jiménez J, et al. Cyclin E amplification/overexpression is a mechanism of trastuzumab resistance in HER2+ breast cancer patients. Proc Natl Acad Sci U S A. 2011; 108: 3761–3766. https://doi.org/10.1073/pnas.1014835108 PMID: 21321214

26. Cocco E, Lopez S, Black J, Bellone S, Bonazzoli E, Predolini F, et al. Dual CCNE1/PIK3CA targeting is synergistic in CCNE1-amplified/PIK3CA-mutated uterine serous carcinomas in vitro and in vivo. Br J Cancer. 2016; 115: 303–311. https://doi.org/10.1038/bjc.2016.198 PMID: 27351214

27. Choudhary GS, Tat TT, Misra S, Hill BT, Smith MR, Almasan A, et al. Cyclin E/Cdk2 dependent phosphorylation of Mcl-1 determines its stability and cellular sensitivity to BH3 mimetics. Oncotarget. 2015; 7: 16912–16925.

28. Xu H, Yu S, Liu Q, Yuan X, Mani S, Pestell RG, et al. Recent advances of highly selective CDK4/6 inhibitors in breast cancer. Journal of Hematology & Oncology. 2017; 10: 97–108.

29. McClue SJ, Blake D, Clarke R, Cowan A, Cummings L, Fischer PM, et al. In vitro and in vivo antitumor properties of the cyclin dependent kinase inhibitor CYC202 (R-roscovitine). Int J Cancer. 2002; 102: 463–468. https://doi.org/10.1002/ijc.10738 PMID: 12432547

30. Cicenas J, Kalyan K, Sorokinas A, Stankunas E, Levy J, Meskinyte I, et al. Roscovitine in cancer and other diseases. Ann Transl Med. 2015; 3:135–146. https://doi.org/10.3978/j.issn.2305-5839.2015.03.61 PMID: 26207228

31. Benson C, White J, De Bono J, O'Donnell A, Raynaud F, Cruickshank C, et al. A phase I trial of the selective oral cyclin-dependent kinase inhibitor seliciclib (CYC202; R-Roscovitine), administered twice daily for 7 days every 21 days. British Journal of Cancer. 2007; 96: 29–37. https://doi.org/10.1038/sj.bjc.6603509 PMID: 17179992

32. Wilson SC, Atrash B, Barlow C, Eccles S, Fischer PM, Hayes A, et al. Design, synthesis and biological evaluation of 6-pyridylmethylaminopurines as CDK inhibitors. Bioorg Med Chem. 2011; 19: 6949–6965. https://doi.org/10.1016/j.bmc.2011.08.051 PMID: 21982796

33. Whittaker SR, Barlow C, Martin MP, Mancusi C, Wagner S, Self A, et al. Molecular profiling and combinatorial activity of CCT068127: a potent CDK2 and CDK9 inhibitor. Mol Oncol. 2018; 12: 287–304. https://doi.org/10.1002/1878-0261.12148 PMID: 29063678

34. Wu SY, McNae I, Kontopidis G, McClue SJ, McInnes C, Stewart KJ, et al. Discovery of a novel family of CDK inhibitors with the program LIDAEUS: structural basis for ligand-induced disordering of the activation loop. Structure. 2003; 11: 399–410. https://doi.org/10.1016/s0969-2126(03)00060-1 PMID: 12679018

35. Wang S, Meades C, Wood G, Osnowski A, Anderson S, Yuill R, et al. 2-Anilino-4-(thiazol-5-yl)pyrimidine CDK inhibitors: synthesis, SAR analysis, X-ray crystallography, and biological activity. J Med Chem. 2004; 47:1662–1675. https://doi.org/10.1021/jm0309957 PMID: 15027857

36. Chou TC, Talalay P. Quantitative analysis of dose-effect relationships: the combined effects of multiple drugs or enzyme inhibitors. Adv Enzyme Regul. 1984; 22: 27–55. https://doi.org/10.1016/0065-2571(84)90007-4 PMID: 6382953

37. Nutley BP, Raynaud FI, Wilson SC, Fischer PM, Hayes A, Goddard PM, et al. Metabolism and pharmacokinetics of the cyclin-dependent kinase inhibitor R-roscovitine in the mouse. Mol Cancer Ther. 2005; 4: 125–139. PMID: 15657360

38. Gojo I, Zhang B, Fenton RG. The cyclin-dependent kinase inhibitor flavopiridol induces apoptosis in multiple myeloma cells through transcriptional repression and down-regulation of Mcl-1. Clin Cancer Res. 2002; 8: 3527–3538. PMID: 12429644

39. Prevo R, Pirovano G, Puliyadi R, Herbert K, Rodriguez-Berriguete G, Docherty O, et al. CDK1 inhibition sensitizes normal cells to DNA damage in a cell cycle dependent manner. Cell Cycle. 2018; 17: 1–11. https://doi.org/10.1080/15384101.2017.1386514

40. Bianchini G, Balko JM, Mayer IA, Sander ME, Gianni L. Triple-negative breast cancer: challenges and opportunities of a heterogeneous disease. Nature Reviews Clinical Oncology. 2016; 13: 674–690. https://doi.org/10.1038/nrclinonc.2016.66 PMID: 27184417

41. Chen X, Low K-H, Alexander A, Jiang Y, Karaka C, Hess KR, et al. Cyclin E Overexpression Sensitizes Triple-Negative Breast Cancer to Wee1 Kinase Inhibition. Clin Cancer Res. 2018; 24: 6594–6610. https://doi.org/10.1158/1078-0432.CCR-18-1446 PMID: 30181387

42. Yin S, Cheryan VT, Xu L, Rishi AK, Reddy KB. Myc mediates cancer stem-like cells and EMT changes in triple negative breast cancers cells. PLoS ONE. 2017; 12(8): e0183578. https://doi.org/10.1371/journal.pone.0183578 PMID: 28817737

43. Poon E, Liang T, Jamin Y, Walz S, Kwok C, Hakkert A, et al. Pharmacological blockade of MYCN in neuroblastoma using orally-bioavailable CDK inhibitors reveals an approach widely applicable to Myc-dependent cancers. J Clin Invest 2020 (revised manuscript under review).

44. Booher RN, Hatch H, Dolinski BM, Nguyen T, Harmonay L, Al-Assaad AS, et al. MCL1 and BCL-xL levels in solid tumors are predictive of dinaciclib-induced apoptosis. PLoS One. 2014; 9: e108371. https://doi.org/10.1371/journal.pone.0108371 PMID: 25289887

45. Blagden S, de Bono J. Drugging cell cycle kinases in cancer therapy. Curr Drug Targets. 2005; 6: 325–335. https://doi.org/10.2174/1389450053765824 PMID: 15857291

46. Senderowicz AM, Headlee D, Stinson SF, Lush RM, Kalil N, Villalba L, et al. Phase I trial of continuous infusion flavopiridol, a novel cyclin-dependent kinase inhibitor, in patients with refractory neoplasms. J Clin Oncol. 1998; 16: 2986–2999. https://doi.org/10.1200/JCO.1998.16.9.2986 PMID: 9738567

47. Kahn ME, Senderowicz A, Sausville EA, Barrett KE. Possible mechanisms of diarrheal side effects associated with the use of a novel chemotherapeutic agent, flavopiridol. Clin Cancer Res. 2001; 7: 343–349. PMID: 11234889

48. Yokoyama A, Lin M, Naresh A, Kitabayashi I, Cleary ML. A higher-order complex containing AF4 and ENL family proteins with P-TEFb facilitates oncogenic and physiologic MLL-dependent transcription. Cancer Cell. 2010; 17: 198–212. https://doi.org/10.1016/j.ccr.2009.12.040 PMID: 20153263

49. Zeisig BB, Milne T, García-Cuéllar MP, Schreiner S, Martin ME, Fuchs U, et al. Hoxa9 and Meis1 are key targets for MLL-ENL-mediated cellular immortalization. Mol Cell Biol. 2004; 24: 617–628. https://doi.org/10.1128/mcb.24.2.617-628.2004 PMID: 14701735

50. Mueller D, García-Cuéllar MP, Bach C, Buhl S, Maethner E, Slany RK. Misguided transcriptional elongation causes mixed lineage leukemia. PLoS Biol. 2009; 7:e1000249. https://doi.org/10.1371/journal.pbio.1000249 PMID: 19956800

51. Raghavan A, Ogilvie RL, Reilly C, Abelson ML, Raghavan S, Vasdewani J, et al. Genome-wide analysis of mRNA decay in resting and activated primary human T lymphocytes. Nucleic Acids Res. 2002; 30: 5529–5538. https://doi.org/10.1093/nar/gkf682 PMID: 12490721

52. Fattman CL, Delach SM, Dou QP, Johnson DE. Sequential two-step cleavage of the retinoblastoma protein by caspase-3/-7 during etoposide-induced apoptosis. Oncogene. 2001; 20: 2918–2926. https://doi.org/10.1038/sj.onc.1204414 PMID: 11420704

53. Letai AG. Diagnosing and exploiting cancer's addiction to blocks in apoptosis. Nat Rev Cancer. 2008; 8: 121–132. https://doi.org/10.1038/nrc2297 PMID: 18202696

54. Zhang H, Guttikonda S, Roberts L, Uziel T, Semizarov D, Elmore SW, et al. Mcl-1 is critical for survival in a subgroup of non-small-cell lung cancer cell lines. Oncogene. 2011; 30:1963–1968. https://doi.org/10.1038/onc.2010.559 PMID: 21132008

55. Do KT, Chau N, Wolanski A, Beardslee B, Hassinger F, Bhushan K, et al. Phase I safety, pharmacokinetic and pharmacodynamic study of CYC065, a cyclin-dependent kinase inhibitor, in patients with advanced cancers (NCT02552953). Proc. Ann. Meeting AACR. 2018; Apr 14–18; Chicago, IL, Abstract CT037.

56. Lu H, Xue Y, Yu GK, Arias C, Lin J, Fong S, et al. Compensatory induction of MYC expression by sustained CDK9 inhibition via a BRD4-dependent mechanism. Elife. 2015; 4:e06535. https://doi.org/10.7554/eLife.06535 PMID: 26083714

57. Campbell KJ, Dhayade S, Ferrari N, Sims AH, Johnson E, Mason SM, et al. MCL-1 is a prognostic indicator and drug target in breast cancer. Cell Death Dis. 2018; 9: 19. https://doi.org/10.1038/s41419-017-0035-2 PMID: 29339815

58. Zervantonakis IK, Iavarone C, Chen H-Y, Selfors LM, Palakurthi S, Liu JF, et al. Systems analysis of apoptotic priming in ovarian cancer identifies vulnerabilities and predictors of drug response. Nature Communications. 2017; 8: 365–377. https://doi.org/10.1038/s41467-017-00263-7 PMID: 28848242

59. Reiner T, de Las Pozas A, Parrondo R, Palenzuela D, Cayuso W, Rai P, et al. Mcl-1 protects prostate cancer cells from cell death mediated by chemotherapy-induced DNA damage. Oncoscience. 2015; 2: 703–715. https://doi.org/10.18632/oncoscience.231 PMID: 26425662

60. Akgul C. Mcl-1 is a potential therapeutic target in multiple types of cancer. Cell Mol Life Sci. 2009; 66: 1326–1236. https://doi.org/10.1007/s00018-008-8637-6 PMID: 19099185

61. Morales F, Giordano A. Overview of CDK9 as a target in cancer research. Cell Cycle. 2016; 15: 519–527. https://doi.org/10.1080/15384101.2016.1138186 PMID: 26766294

62. Xiang W, Yang C-Y, Longchuan Bai L. MCL-1 inhibition in cancer treatment. Onco Targets Ther. 2018; 11: 7301–7314. https://doi.org/10.2147/OTT.S146228 PMID: 30425521

63. Monroe SC, Jo SY, Sanders DS, Basrur V, Elenitoba-Johnson KS, Slany RK, et al. MLL-AF9 and MLL-ENL alter the dynamic association of transcriptional regulators with genes critical for leukemia. Exp Hematol. 2011; 39: 77–86.e1-5. https://doi.org/10.1016/j.exphem.2010.09.003 PMID: 20854876

64. McDonald ER 3rd, de Weck A, Schlabach MR, Billy E, Mavrakis KJ, Hoffman GR, et al. Project DRIVE: A Compendium of Cancer Dependencies and Synthetic Lethal Relationships Uncovered by Large-Scale, Deep RNAi Screening. Cell. 2017; 170(3): 577–592.e10. https://doi.org/10.1016/j.cell.2017.07.005 PMID: 28753431

65. De Leon G, Cavino M, D'Angelo M, Krucher NA. PNUTS knockdown potentiates the apoptotic effect of Roscovitine in breast and colon cancer cells. Int J Oncol. 2010; 36: 1269–1275. https://doi.org/10.3892/ijo_00000611 PMID: 20372802

66. Lentine B, Antonucci L, Hunce R, Edwards J, Marallano V, Krucher NA. Dephosphorylation of threonine-821 of the retinoblastoma tumor suppressor protein (Rb) is required for apoptosis induced by UV and Cdk inhibition. Cell Cycle. 2012; 11: 3324–3330. https://doi.org/10.4161/cc.21693 PMID: 22895174

67. Schraml P, Bucher C, Bissig H, Nocito A, Haas P, Wilber K, et al. Cyclin E overexpression and amplification in human tumours. J Pathol. 2003; 200: 375–382. https://doi.org/10.1002/path.1356 PMID: 12845634

68. Min A, Kim JE, Kim Y-J, Lim JL, Kim S, Kim JW, et al. Cyclin E overexpression confers resistance to the CDK4/6 specific inhibitor palbociclib in gastric cancer cells. Cancer Letters Volume 430, 28 August 2018, pp 123–132. https://doi.org/10.1016/j.canlet.2018.04.037 PMID: 29729292

69. Turner NC, Liu Y, Zhu Z, Loi S, Colleoni M, Loibl S, et al. Cyclin E1 expression and palbociclib efficacy in previously treated hormone receptor–positive metastatic breast cancer. JCO. 2019; https://doi.org/10.1200/JCO.18.00925

70. Herrera-Abreu MT, Palafox M, Asghar U, Rivas MA, Cutts RJ, Garcia-Murillas I, et al. Early Adaptation and Acquired Resistance to CDK4/6 Inhibition in Estrogen Receptor-Positive Breast Cancer. Cancer Res. 2016; 76: 2301–2313. https://doi.org/10.1158/0008-5472.CAN-15-0728 Erratum in: Cancer Res. 2016; 76: 5907. PMID: 27020857

71. Doostan I, Karakas C, Kohansal M, Low KH, Ellis MJ, Olson JA Jr, et al. Cytoplasmic Cyclin E Mediates Resistance to Aromatase Inhibitors in Breast Cancer. Clin Cancer Res. 2017; 23: 7288–7300. https://doi.org/10.1158/1078-0432.CCR-17-1544 PMID: 28947566

72. Meyers RB, Bryan JG, McFarland JM, Weir BA, Sizemore AE, Xu H, et al. Nature Genetics, 2017; 49: 1779–1784. https://doi.org/10.1038/ng.3984 PMID: 29083409
